# Bone-Targeting Microspheres Enable Sustained Release of CD301b^+^ Macrophage-Derived Small Extracellular Vesicles to Promote Bone Repair

**DOI:** 10.7150/thno.132666

**Published:** 2026-06-17

**Authors:** Xiang Gao, Shanshan Ma, Yeying Dong, Fengkai Yang, Rui Huang, Xiudan Zheng, Zhijun Liu, Hong Zheng, Fatai Lu, Thomas Groth, Mingyan Zhao

**Affiliations:** 1 Stem Cell Research and Cellular Therapy Center, Affiliated Hospital of Guangdong Medical University, Zhanjiang 524001, China.; 2 Orthopedic Center, Affiliated Hospital of Guangdong Medical University, Zhanjiang 524001, China.; 3 Department Biomedical Materials, Institute of Pharmacy, Martin Luther University Halle-Wittenberg, 0699 Halle (Saale), Germany.; 4 Guangdong Provincial Key Laboratory of Autophagy and Major Chronic Non-communicable Diseases, Affiliated Hospital of Guangdong Medical University, Zhanjiang 524001, China.

**Keywords:** bone repair, hydrogel microsphere, CD301b⁺ macrophage-derived small extracellular vesicles, targeted release, multifunctional synergy

## Abstract

**Rationale:**

Large bone defects often exceed the body’s intrinsic capacity for self-repair. Successful bone healing depends on a coordinated immune transition alongside tightly coupled angiogenesis and osteogenesis, in which macrophages play a key regulatory role. Therefore, ideal bone-regenerative materials should integrate immunomodulatory, pro-angiogenic, and osteogenic functions.

**Methods and Results:**

Small extracellular vesicles (sEVs) from CD301b^+^ macrophages (CD301b⁺-sEVs) are isolated by ultracentrifugation for inclusion in a bone-targeting microsphere system fabricated by microfluidic technology through dynamic Schiff-base cross-linking of alendronate-functionalized succinylated chitosan with oxidized sodium alginate allowing pH-responsive release of CD301b⁺-sEVs. Transcriptome sequencing and comprehensive *in vitro* studies reveal that CD301b⁺-sEVs possess the ability to modulate immune homeostasis by promoting macrophage polarization toward the pro-repair M2 phenotype, upregulate angiogenic factors that can enhance vascularization as seen by HUVEC sprouting assay, and contain RNA that stimulate osteogenic differentiation of BMSCs through activation of the Akt/GSK-3β/β-catenin signaling pathway. Preclinical studies with a rat calvaria model show that microspheres loaded with CD301b^+^-sEVs significantly promote bone regeneration in comparison to microsphere controls.

**Conclusion:**

This study not only overcomes key limitations of conventional sEVs therapies such as poor retention and instability, but also provides an innovative “targeted localization, intelligent release, and multifunctional synergy” strategy, offering a minimally invasive and highly effective therapeutic platform for bone repair.

## Introduction

Bone defects caused by trauma, surgery, tumors, or infections often require clinical intervention when the damage exceeds the intrinsic healing capacity of bone 1. Although autologous bone grafting remains the current gold standard, its clinical use is limited by donor site morbidity, restricted tissue availability, and the risk of infection or secondary injury 2. Therefore, there is a continued need to develop more effective materials for bone repair. Bone repair is a tightly orchestrated process that includes sequential inflammatory, fibrovascular, and bone formation/remodeling phases 3. Successful repair depends not only on timely resolution of inflammation, but also on the coordination of angiogenesis and osteogenesis 4. From this perspective, biomaterials designed for bone regeneration should ideally provide immunomodulatory, angiogenic, and osteogenic support within the defect microenvironment.

Macrophages are key regulators of the local microenvironment during bone regeneration 5. Because of their functional plasticity, they have attracted increasing attention as potential therapeutic targets in bone repair. In general, M1 macrophages are associated with the initiation and amplification of inflammation, whereas M2 macrophages contribute to inflammation resolution, vascularization, and tissue repair 6-8. However, the biological functions of macrophages in tissue repair cannot be fully explained by the simple M1/M2 classification. Increasing evidence suggests that specific macrophage subsets may play more direct roles in regeneration. Among these subsets, CD301b⁺ macrophages have recently been identified as a distinct reparative population that partially overlaps with conventional polarization markers but exhibit unique immunomodulatory and pro-regenerative properties 9, 10.* In vitro* studies have shown that CD301b^+^ macrophages promote osteogenesis of mesenchymal stem cells (MSCs) 11 and enhance angiogenesis 12.* In vivo* transplantation of limited numbers of CD301b^+^ macrophages significantly improves the osteoinductive performance of biphasic calcium phosphate ceramics. By contrast, selective depletion of CD301b^+^ macrophages in *Mgl2*^DTR^ mice markedly reduces bone formation, further supporting their essential role in bone homeostasis 11. Together, these findings indicate that CD301b⁺ macrophages are important regulators of the microenvironment during bone repair and may provide a useful basis for the design of multifunctional regenerative biomaterials. Despite their therapeutic promise, direct cell delivery remains challenging because of poor cell survival and retention after transplantation, difficulty in dose control, and limited spatial precision of delivery 13. These limitations restrict the efficient application of exogenous CD301b⁺ macrophages at bone defect sites.

Currently, cell-derived small extracellular vesicles (sEVs, 30-200 nm in diameter), have rapidly emerged as promising cell-free agents in regenerative medicine, owing to their safety, efficacy, and ready availability. These vesicles encapsulate a diverse array of bioactive substances (e.g., proteins and nucleic acids) from their parental cells, which are internalized by recipient cells to exert biological functions 14. It is well documented that sEVs are key mediators for intercellular communication. Recent studies reveal that macrophages primarily regulate bone homeostasis through sEVs secretion 15. Zhou and the coworkers 16 reported that sEVs derived from M2 macrophages (M2-sEVs) can promote the osteogenic differentiation of MSCs, while more recent research indicates that M2-sEVs also function as immune regulators to facilitate fracture healing, mirroring the biological function of M2 macrophages 17. sEVs inherit functional properties from their source cells which are further supported by studies on stem cell-derived sEVs. For instance, sEVs from MSCs have been shown to recapitulate the therapeutic effects of MSCs in tissue regeneration and immunomodulation 18. Therefore, it can be expected that sEVs from CD301b^+^ macrophages (CD301b^+^-sEVs) will retain their parent cells’ immunomodulatory, pro-angiogenic, and osteogenic abilities, making them promising candidates for cell-free bone healing therapies. Importantly, using sEVs avoids the potential immunological risks associated with direct administration of CD301b⁺ macrophages, which could otherwise trigger inflammatory responses in recipients. However, because sEVs are typically administered by injection and degrade quickly in liquid form, their retention and action at the defect sites is limited 19.

Hydrogel microspheres can be used to fill irregular bone defects by minimal invasive procedures, enabling precise delivery of drugs or osteogenic factors including sEVs 20 to the target site with local effective concentrations and long-term effects 21. Highly monodisperse microspheres can be fabricated through microfluidics, enabling a one-step encapsulation of drugs or cells 22. Hydrophilic polysaccharides like chitosan (CH) and sodium alginate (SA) with well-document biocompatibility, low immunogenicity can be used for production of hydrogel microspheres 23, 24. Because of the reactive amino groups in CH, solubility of CH at physiological pH can be achieved by succinylation (SCH), while aldehyde moieties can be introduced by oxidizing SA (oxidized SA, OSA) for cross-linking reactions with amino groups in SCH 25. These modifications enable reversible cross-linking of SCH and OSA via Schiff base bonds 26, forming a pH-responsive three-dimensional hydrogel microspheres network. For effective bone defect treatment, microspheres require precise localization and long-term retention at the defect site which can be achieved by alendronate (ALE) exhibiting high affinity for hydroxyapatite (HAP) 27. Leveraging the amino group within ALE, aldehyde-activated ALE can be grafted onto SCH through Schiff base reactions 28, forming the bone-targeting composite carrier ALE-SCH (named A-SCH).

**Scheme 1:**
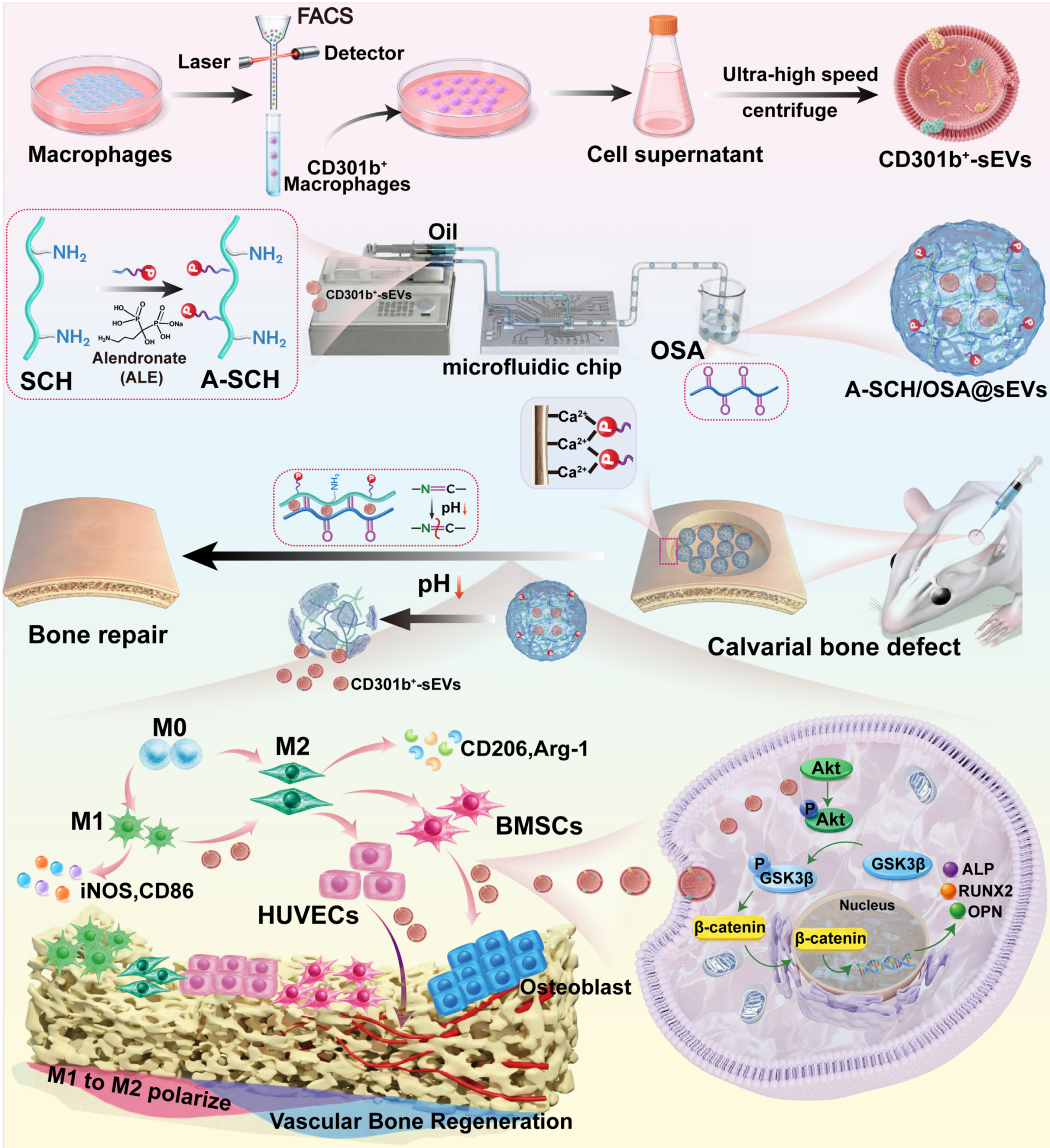
Schematic illustration of pH-responsive bone-targeting microspheres loaded with CD301b^+^-sEVs promoting bone regeneration for orchestration of immune, osteogenic, and angiogenic processes.

We aimed to develop here an innovative multifunctional microsphere system by integrating CD301b^+^-sEVs into microspheres composed of A-SCH and OSA (**Scheme 1**). For the first time, high-purity CD301b^+^-sEVs were isolated using fluorescence-activated cell sorting (FACS) coupled with ultracentrifugation and characterized by transcriptome sequencing and comprehensive* in vitro* comparative analyses with conventional M2-sEVs, studying their ability to modulate macrophage polarization, promote angiogenesis, and drive osteogenic differentiation. Then, we pioneered to embed CD301b^+^-sEVs in A-SCH/OSA monodisperse, injectable, pH-responsive, and bone-targeting microspheres fabricated via microfluidic technology (designated as A-SCH/OSA@sEVs) to achieve precise localization and extended retention at bone defect sites, facilitating targeted and controlled release of CD301b^+^-sEVs. By conducting comparative studies with sEVs-free microspheres (A-SCH/OSA), we systematically evaluated the biological effects elicited by A-SCH/OSA@sEVs in cell culture and in a rat calvarial defect model to achieve a coordinated regulation of immune, vascular, and skeletal processes. Moreover, the underlying mechanism of CD301b^+^-sEVs-mediated osteogenesis was elucidated. Collectively, this work summarized in **Scheme 1** not only provides a promising cell-free therapeutic platform for repair of bone defects but also significantly advances the translation potential of sEVs-based regenerative medicine by leveraging the unique bioactivity of CD301b⁺-sEVs.

## Materials and Methods

### Materials

Chitosan (CH), lipopolysaccharides (LPS), L-ascorbic acid, dexamethasone, β-sodium glycerophosphate and PKH26 red fluorescent cell linker mini kit were supplied by Sigma (USA). Sodium alginate (SA) was ordered from Aladdin (China). The live/dead staining kit was purchased from Proteintech (China). Interleukin-4 (IL-4) was purchased from PeproTech (USA). Fetal bovine serum (FBS), alpha-modified Eagle’s medium (α-MEM), penicillin-streptomycin, and trypsin were obtained from Gibco (USA). High glucose Dulbecco’s modified eagle medium (DMEM) was ordered from Wuhan Procell Life Science & Technology Co., Ltd. (China). Cell counting kit-8 (CCK8) was obtained from Zeta Life (USA). BCA protein assay kit was ordered from Beyotime (China). LY294002 was purchased from MCE (USA). All primers were obtained from Sangon Biotechnology Co., Ltd. (China). Sprague-Dawley (SD) rats were provided by SiPeiFu Biotechnology Co., Ltd. (China). The polydymethylsiloxane (PDMS) microfluidic chip (channel dimensions: 125 μm×125 μm) was purchased from Shenzhen Huanova Biotechnology Co., Ltd. (China).

### Cell culture

The murine macrophage cell line RAW264.7 was provided by Wuhan Procell Life Science&Technology Co., Ltd., while the human umbilical vein endothelial cell (HUVEC) line was a generous gift from Dr. Qingyu Zhang (Department of Obstetrics and Gynecology, Affiliated Hospital of Guangdong Medical University). Both RAW264.7 cells and HUVECs were grown in DMEM containing 10% FBS and 1% penicillin-streptomycin. Human BMSCs were isolated and characterized following an established protocol, with detailed methods provided in the **Supplementary Materials**. BMSCs were grown in α-MEM complete medium with 10% FBS and 1% penicillin-streptomycin, and all cell lines were kept at 37 °C in a humidified 5% CO₂ atmosphere.

### Isolation, characterization and transcriptome sequencing of sEVs

RAW264.7 cells were pretreated with 20 ng/mL IL-4, and then CD301b^+^ macrophages were sorted *in vitro* based on an established protocol 11. To isolate sEVs, supernatants from CD301b^-^, CD301b^+^ macrophages or M2-polarized macrophages were collected and subjected to a series of ultracentrifugation and filtration steps, following established protocols 29. Details of macrophages sorting, sEVs isolation and characterization are provided in the **Supplementary Materials**. To analyze the differential expression of transcriptomic profiles between the CD301b⁻-sEVs and CD301b⁺-sEVs, whole transcriptome sequencing was performed by Beijing Allwegene Technology Company Limited (China). Differentially expressed genes (DEGs) were identified and subjected to Gene Ontology (GO) enrichment and KEGG pathway analyses. Details procedures for transcriptome sequencing and data analysis are provided in the **Supplementary Materials**.

### Uptake of sEVs by BMSCs, HUVECs, and RAW264.7

CD301b^+^-sEVs and M2-sEVs were fluorescently labeled using the PKH26 kit following the manufacturer’s instruction, detailed methods are presented in the **Supplementary Materials.** To track sEVs uptake, recipient cells (BMSCs, HUVECs or RAW264.7 cells) were incubated with 40 μg/mL PKH26-labeled sEVs (CD301b^+^-sEVs and M2-sEVs) for 24 h. Additionally, to investigate the time-dependent uptake kinetics specifically for CD301b^+^-sEVs, the recipient cells were incubated with 40 μg/mL PKH26-labeled CD301b^+^-sEVs for 6, 12, and 24 h. Following incubation at the respective time points, cells were then washed with PBS, fixed with 4% paraformaldehyde (15 min), and counterstained with DAPI (Invitrogen, USA) to label nuclei. sEVs internalization was visualized under a confocal laser scanning microscope (CLSM, FV3000, Olympus, Japan) and images were analyzed by Image J software (version 1.51j8).

### Preparation of microspheres

SCH, OSA and A-SCH were synthesized and characterized as described in the **Supplementary Materials**. Monodisperse microspheres were fabricated using microfluidic technology, with CD301b^+^-sEVs incorporated in their unlabeled form unless otherwise stated. Briefly, the aqueous phase, consisted either of 10 mg/mL SCH, 10 mg/mL A-SCH, or 10 mg/mL A-SCH containing 0.5 mg/mL CD301b^+^-sEVs. The oil phase was composed of mineral oil with 5% (v/v) Span 80. Both fluids were separately injected into the microfluidic chip at controlled flow rates of 2 μL/min (aqueous phase) and 20 μL/min (oil phase), respectively. The resulting emulsion droplets were then collected in a 30 mg/mL OSA solution and crosslinked via Schiff base reaction on a shaker at 4 °C for 12 h. The droplets were then washed with anhydrous ethanol to remove residual oil and rinsed with deionized water. After centrifugation at 3,500 rpm for 5 min, the purified microspheres were collected for subsequent studies. The microspheres were denoted as SCH/OSA, A-SCH/OSA, and A-SCH/OSA@sEVs, respectively.

### Characterization of microspheres

Various analytical methods were used to examine the structure and morphology of the microspheres. Microsphere morphology was examined using an inverted fluorescence microscope (IX73, Olympus). Particle diameters were statistically analyzed (≥200 microspheres/group) using Nano Measure software (version 1.2.5), with size distribution histograms generated using Origin software (version 2019b). The surface morphology of air-dried microspheres was observed by scanning electron microscopy (SEM, Regulus8100, Hitachi, Japan). The chemical composition was detected by Fourier transform infrared spectroscopy (FT-IR, Nicolet iS50, Thermo Scientific).

### HAP affinity assay

An *in vitro* HAP affinity assay was conducted to evaluate the bone targeting capacity of microspheres. Briefly, a HAP disk (diameter of 11 mm and thickness of 3 mm, Aladdin, China) was placed in each well of a 24-well plate (Labselect, China). Equal amounts of SCH/OSA microspheres (non-targeted) and A-SCH/OSA microspheres (targeted) were dispersed in 0.5 mL 0.9% NaCl and added to wells containing HAP disks (three replicate per sample). The plate was incubated on an orbital shaker. After 4, 14, and 24 h of incubation, HAP disks were retrieved and gently rinsed with 0.9% NaCl to remove unbound microspheres. Adherent microspheres on the disks were then observed and imaged using an inverted fluorescence microscope. ImageJ software (version 1.53k) was used to quantify the bound microspheres.

### sEVs encapsulation in microspheres

To confirm the successful encapsulation of CD301b^+^-sEVs, DIR-labeled CD301b^+^-sEVs (sEVs^DIR^) were first prepared and then encapsulated using a microfluidic technique. The resulting A-SCH/OSA@sEVs^DIR^ microspheres were cryosectioned into 5.0 μm-thick specimens. The cryosections were spun coated with platinum-palladium using the Ion Sputter E-100 coating system (Hitachi High-Technologies) and observed by SEM at an accelerating voltage of 3 kV. Meanwhile, the spatial distribution of the encapsulated sEVs was examined by CLSM.

### sEVs release from microspheres

To evaluate the release kinetics of sEVs from microspheres, equal aliquots of A-SCH/OSA@sEVs microspheres were immersed in 300 µL of simulated body fluid (SBF, pH = 7.4 or 5, Lengen, China) and subsequently placed in a shaker maintained at 37 ℃ and 100 rpm. On days 1, 3, 5, 7, 9, 11, 13, and 15, 20 µL of supernatant were withdrawn and immediately replenished with 20 µL of fresh SBF (pH = 7.4 or 5). CD301b^+^-sEVs content in each collected sample was measured by the BCA method. The cumulative amount of sEVs released was determined using the following formulation (1).



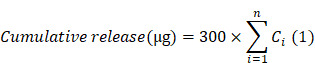



where* C_i_* represents the concentration of sEVs released at the respective sampling time point.

On day 15, the microspheres incubated in the respective release media were collected and air-dried at room temperature and then examined under SEM to observe their surface morphology and structural features.

### Osteogenic differentiation and evaluation

The biocompatibility of the microspheres was evaluated separately, and detailed experimental procedures are described in the **Supplementary Materials**. For osteogenic induction, BMSCs were seeded at 1×10^5^ cells per well in α-MEM complete medium in 6-well plates. Upon reaching 80% confluence, the cells were switched to osteogenic induction medium (OIM), consisting of α-MEM supplemented with 10% FBS, 1% penicillin-streptomycin, 50 μM L-ascorbic acid, 10 mM β-sodium glycerophosphate, and 100 nM dexamethasone. Cells were divided into the following treatments: OIM alone (control), OIM with sEVs (M2-sEVs or CD301b^+^-sEVs at 40 μg/mL), or OIM with microspheres (SCH/OSA, A-SCH/OSA, or A-SCH/OSA@sEVs at 2 mg per well). The medium was changed every 3 days.

After 6 days of induction, the cells were gently rinsed with PBS and then fixed in 4% paraformaldehyde for 15 min. ALP activity was then assessed using a BCIP/NBT ALP Color Development Kit (Beyotime) following the manufacturer’s instructions. Stained samples were visualized using an inverted microscope. In parallel, total RNA and protein lysates were harvested from cells after 6 days of induction. The mRNA expression levels of osteogenic markers (RUNX2, ALP, OPN) were quantified by RT-qPCR with GAPDH as the internal reference, and the corresponding protein levels were analyzed by Western blot, detailed experimental procedures are described in the **Supplementary Materials**. After 12 days of induction, mineralization was assessed using Alizarin Red S (ARS) staining (OriCell, China). Cells were fixed and incubated with ARS solution for 15 minutes. Images of stained samples were taken using an inverted microscope. To quantify calcium deposition, ARS-stained nodules were dissolved in 10% (w/v) 1-cetylpyridinium chloride monohydrate (Sigma), and absorbance was measured at 562 nm using a multifunctional microplate reader.

### Immunomodulatory effect assessment

RAW264.7 cells were plated onto sterile glass coverslips inside 24-well plates overnight and were driven toward the M1 phenotype by adding 100 ng/mL LPS for 12 h 30. After M1 polarization inducement, the cells were further treated for 48 h under the following conditions: (1) direct treatment: sEVs (M2-sEVs or CD301b⁺-sEVs at 40 μg/mL) were added directly to the complete medium. (2) Transwell co-culture: various microspheres (SCH/OSA, A-SCH/OSA, or A-SCH/OSA@sEVs, 0.5 mg per well) were loaded into the upper compartment of a Transwell. Cells cultured in normal medium without LPS stimulation served as the control. Following treatment, cells were sequentially fixed (4% paraformaldehyde), permeabilized (0.1% Triton X-100) and blocked (1% BSA), then kept overnight at 4 °C with primary antibodies targeting iNOS (1:200, Zenbio), or CD206 (1:200, Cell signaling Technology, USA). Thereafter, the cells were treated with Alexa Fluor 488- or Alexa Fluor 568-labeled goat anti-rabbit IgG (both 1:400, Invitrogen), along with DAPI for nuclear labeling. Images were taken with a CLSM and then processed using ImageJ software. RT-qPCR was further used to determine the expression of M1 proinflammatory genes (CD86, IL-1β) and M2 anti-inflammatory genes (CD206 and Arg-1) in RAW264.7 cells.

### Angiogenic activity evaluation

Proangiogenic effects were evaluated using tube formation assays in two distinct experimental systems. For direct sEVs treatment, HUVECs (3 × 10^5^ cells per well) were placed in Matrigel-coated 24-well plates and incubated with M2-sEVs or CD301b⁺-sEVs (40 μg/mL). Cells cultured in normal medium without added sEVs served as the control. To assess microsphere-mediated (indirect) angiogenesis modulation, a Transwell co-culture system (24-well plate, 8 μm pore) was established. Microspheres (SCH/OSA, A-SCH/OSA, or A-SCH/OSA@sEVs, 0.5 mg per well) suspended in FBS free DMEM were loaded into upper chambers, while HUVECs (3 × 10^5^ cells per well) were plated onto Matrigel-coated lower chambers. Following 6 h of incubation in both systems, calcein-AM (Proteintech) was used to stain the cells. The stained cells were then visualized by inverted fluorescence microscopy, and the tube formation parameters were quantified using Angio Tool (version 0.6 a).

For molecular characterization, HUVECs were plated into 6-well plates. After adhesion, the culture medium was exchanged with complete medium under the following conditions: supplemented with sEVs (M2-sEVs or CD301b^+^-sEVs, 40 μg/mL) or with various microspheres (SCH/OSA, A-SCH/OSA, or A-SCH/OSA@sEVs, 2 mg per well). Cells cultured in complete medium without added sEVs or microspheres served as the control. The transcript levels of angiogenesis-associated genes (VEGF, CD31, and eNOS) were quantified by RT-qPCR, and protein expression levels of angiogenesis-related markers (VEGF, HIF-1α, and CD31) were evaluated by Western blot analysis.

### Animal experiments

Full-thickness calvarial defects of 5 mm in diameter were generated along the midline of the skull in anesthetized SD rats using an established methodology 31. For* in vivo* targeting evaluation, male rats (6 weeks old, 170~190 g) were implanted with either A-SCH/OSA^Cy5.5^ or SCH/OSA^Cy5.5^ microspheres at a dose of 10 mg per defect (suspended in 100 μL saline). These microspheres were prepared by incorporating the near-infrared fluorescent dye Cy5.5-NHS (Lumiprobe, USA) during fabrication to enable *in vivo* optical imaging 32. The fluorescence intensity at the implantation site was monitored at 0, 1-, 3-, and 5-days post-surgery using the Ami HTX system for *in vivo* imaging (Spectral Instruments Imaging, USA) and quantified with Aura 4.0 software.

For bone regeneration studies, male rats (6 weeks old, 170~190 g) were randomly assigned to four groups: defect-only (control, no microsphere implantation), SCH/OSA, A-SCH/OSA, and A-SCH/OSA@sEVs. Except for the control group, each defect received 10 mg of the respective microspheres (suspended in 100 μL of saline). The control group was injected with 100 μL of saline (vehicle). At 10 weeks post-surgery, all animals were euthanized. Calvariae and major organs were harvested for subsequent analysis.

### Micro-CT analysis

Rat skulls were kept in 4 % paraformaldehyde for 48 h prior to micro-CT analysis. Three-dimensional (3D) imaging was carried out with a high-resolution *in vivo* micro-CT scanner (viva CT80, SCANCO Medical AG, Switzerland). Bone mineral density (BMD), the ratio of bone volume to total volume (BV/TV), trabecular number (Tb.N), and trabecular thickness (Tb.Th) in the defect site were quantified using CTAn Analyzer software (Version 1.13, Bruker Micro-CT, Kontich, Belgium). Then, CTvox software (Bruker Micro-CT) was used to reconstruct 3D and 2D micro-CT images.

### Histological and immunohistochemical fluorescence assessment

After micro-CT examination, all rat skull samples were decalcified in 12% EDTA buffer solution (pH 7.4, Solarbio) for 4 weeks, with solution refreshed twice a week. Subsequently, the decalcified rat skull samples, along with the pre-fixed internal organs were paraffin-embedded and sliced into 5-μm sections. To evaluate new bone formation, hematoxylin and eosin (H&E, Solarbio) staining and Masson’s trichrome staining were performed, and the stained sections were imaged using a microscope (Bx53, Olympus). Immunohistochemical fluorescence staining was performed to detect ALP, RUNX2, OCN (Zenbio), CD31, VEGF, α-SMA (Proteintech), CD206, CD86 (Zenbio), iNOS, TNF-α (Proteintech), and IL-10 (Proteintech). All antibodies were diluted at a ratio of 1:200. All stained sections were imaged using a CLSM (LSM900, Zeiss, Germany).

### Statistical analysis

All data were obtained from at least three independent samples and are presented as means ± standard deviation. All statistical calculations were carried out using SPSS 19.0. To compare two groups, we used Student’s t-test; for multiple comparisons, one-way ANOVA followed by Tukey’s post hoc test was performed. The levels of statistical significance are defined as: ^*^P < 0.05, ^**^P < 0.01, and ^***^P < 0.001.

## Results and Discussion

### CD301b^+^-sEVs: sorting, characterization and transcriptome sequencing

**Figure 1:**
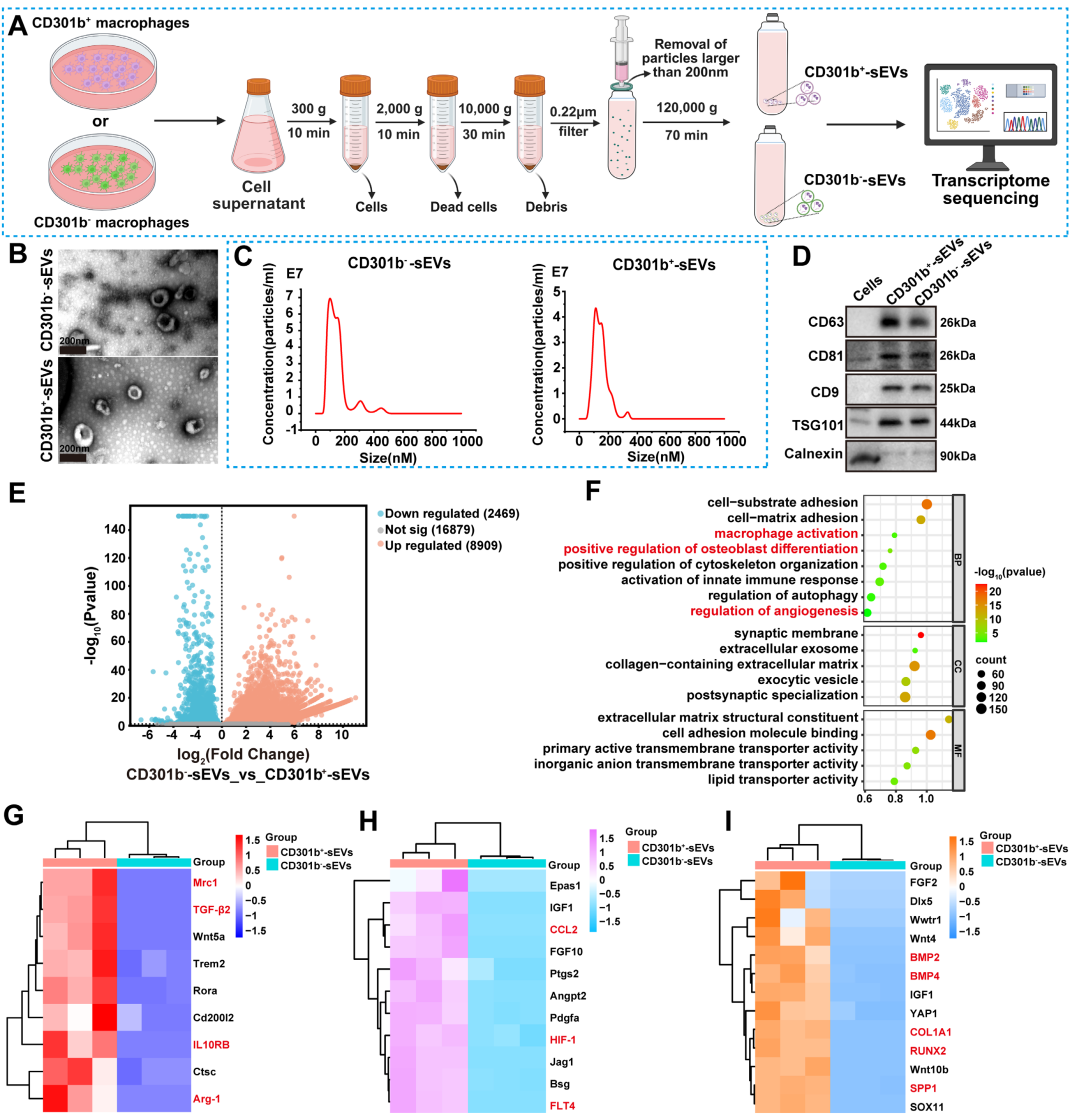
Characterization and transcriptome sequencing of CD301b⁻-sEVs and CD301b⁺-sEVs. (A) Schematic of differential ultracentrifugation process for isolating CD301b⁻-sEVs and CD301b⁺-sEVs. (B) Representative TEM images of CD301b⁻-sEVs and CD301b⁺-sEVs. (C) Size distribution of CD301b⁻-sEVs and CD301b⁺-sEVs determined by NTA. (D) Western blot analysis of sEV positive markers (CD63, CD81, CD9, and TSG101) and negative control (Calnexin) in RAW264.7 cells, CD301b⁻-sEVs, and CD301b⁺-sEVs. (E) Volcano plot of DEGs, upregulated (red), downregulated (green), and non-significantly changed genes (gray), respectively. (F) GO enrichment analysis of upregulated DEGs in CD301b⁺-sEVs compared with CD301b⁻-sEVs. (G) Heatmap of macrophage polarization-related gene expression (red: high, blue: low). (H) Heatmap showing expression of genes associated with angiogenesis (I) Heatmap of osteogenesis-related gene expression. **Figure 1A** was generated using BioRender, and the publishing and licensing rights were confirmed under agreement number QB28SW4EN3.

To investigate the functional roles and underlying mechanisms of CD301b^+^-sEVs in promoting bone formation, we first isolated CD301b^+^ and CD301b^-^ macrophage subsets from IL-4-stimulated RAW264.7 cells using FACS **(Figure S1A)**. Quantitative analysis showed that CD301b⁺ macrophages accounted for approximately 40.7% of the isolated cells **(Figure S1B)**, which is consistent with previous reports 11. sEVs were then isolated from the culture supernatants by differential ultracentrifugation, yielding CD301b^-^-sEVs and CD301b^+^-sEVs** (Figure 1A)**. TEM showed that both vesicle populations displayed the typical cup-shaped morphology and bilayer membrane structure of sEVs **(Figure 1B)**. NTA analysis further showed that the vesicles ranged from 70 to 200 nm in diameter, with a peak around 100 nm **(Figure 1C)**, consistent with the expected size distribution of sEVs 25. The presence of typical sEV markers CD9, CD81, CD63, and TSG101 was verified by Western blotting, while the negative marker calnexin was barely detected in both sEVs** (Figure 1D)**. These results indicate that sEVs were successfully isolated from both CD301b⁻ and CD301b⁺ macrophages.

To compare the molecular features of CD301b⁻-sEVs and CD301b⁺-sEVs, we next performed transcriptome sequencing. Clear differences in gene expression were observed between the two groups. Relative to CD301b⁻-sEVs, CD301b⁺-sEVs exhibited 8,909 upregulated genes and 2,469 downregulated genes (|log₂ FC| > 1, *P* < 0.05). **(Figure 1E)**. GO enrichment analysis of these DEGs identified several biological processes that were significantly enriched in CD301b⁺-sEVs **(Figure 1F)**. Among the immune-related pathways, macrophage activation was significantly enriched (*P* = 0.0019), involving 35 genes, including *Mrc1 (CD206), TGF-β2, Wnt5a, IL-10RB, Trem2, Rora, Cd200l2, Ctsc* and* Arg-1* **(Figure 1G).** Angiogenesis-related processes were also enriched (*P* = 0.007), with upregulation of genes such as *Epas1, IGF-1*, *FGF10, Ptgs2*, *angpt2*, *pdgfa*, *HIF-1, CCL2*, *Jag1*, *Bsg*, and* FLT4 (VEGFR-3*) (**Figure 1H)**. In addition, positive regulation of osteoblast differentiation was one of the most significantly enriched categories (*P* = 0.00001), including 33 genes such as *FGF2, Dlx5, Wwtr1, Wnt4, BMP2, BMP4, IGF1, YAP1, COL1A1, RUNX2, Wnt10b, SPP1* and* SOX11* **(Figure 1I)***.* Overall, these transcriptomic data suggest that CD301b⁺-sEVs have stronger potential to regulate inflammation, support angiogenesis, and promote osteogenic differentiation than CD301b⁻-sEVs. Previous studies have shown that CD301b⁺ macrophages possess greater osteogenic activity than CD301b⁻ macrophages 11, 33. Our results further suggest that this pro-regenerative profile may be retained in their secreted sEVs, highlighting the potential of CD301b⁺-sEVs as a cell-free strategy for bone repair.

### Validation of multiple effects of CD301b^+^-sEVs *in vitro*

Previous evidence demonstrates multifunctionality of M2-sEVs, including their ability to actively regulate immune microenvironment, induce osteogenic differentiation of MSCs 16, and exerting potent pro-angiogenic capabilities 34. Thus, M2-sEVs were successfully isolated (**Figure S2A-C**) and served as a positive control to investigate the multiple effects of CD301b^⁺^-sEVs. As cellular internalization is the first step by which sEVs exert regulatory functions on recipient cells, we first investigated the uptake of CD301b⁺-sEVs and M2-sEVs. After 24 h of co-culture, both types of sEVs were effectively internalized by RAW264.7 cells, HUVECs, and BMSCs **(Figure S4A, B)**. To further characterize the uptake kinetics of CD301b⁺-sEVs, we examined their internalization at 6, 12, and 24 h. As shown in **Figure S5A**, uptake increased progressively with time in all three cell types, with the most pronounced increase observed between 12 and 24 h. Quantitative fluorescence analysis revealed that BMSCs and HUVECs exhibited higher total uptakes than RAW264.7 cells, with BMSCs showing the greatest fluorescence intensity** (Figure S5B)**. These results indicate that CD301b⁺-sEVs are internalized by all three reparative cell populations, with a particularly strong uptake capacity in endothelial and osteoprogenitor cells. It is important to note that direct comparison of uptake efficiency across different cell types is confounded by differences in cell size and culture conditions; moreover, the complex and dynamic nature of the *in vivo* bone defect microenvironment precludes any direct extrapolation of* in vitro* uptake kinetics to a temporal hierarchy* in vivo*. Consequently, our study does not claim a temporal order of cellular targeting. Instead, these data support the concept that CD301b⁺-sEVs can act on multiple target cells concurrently, providing a cellular basis for their coordinated immunomodulatory, angiogenic, and osteogenic effects.

**Figure 2:**
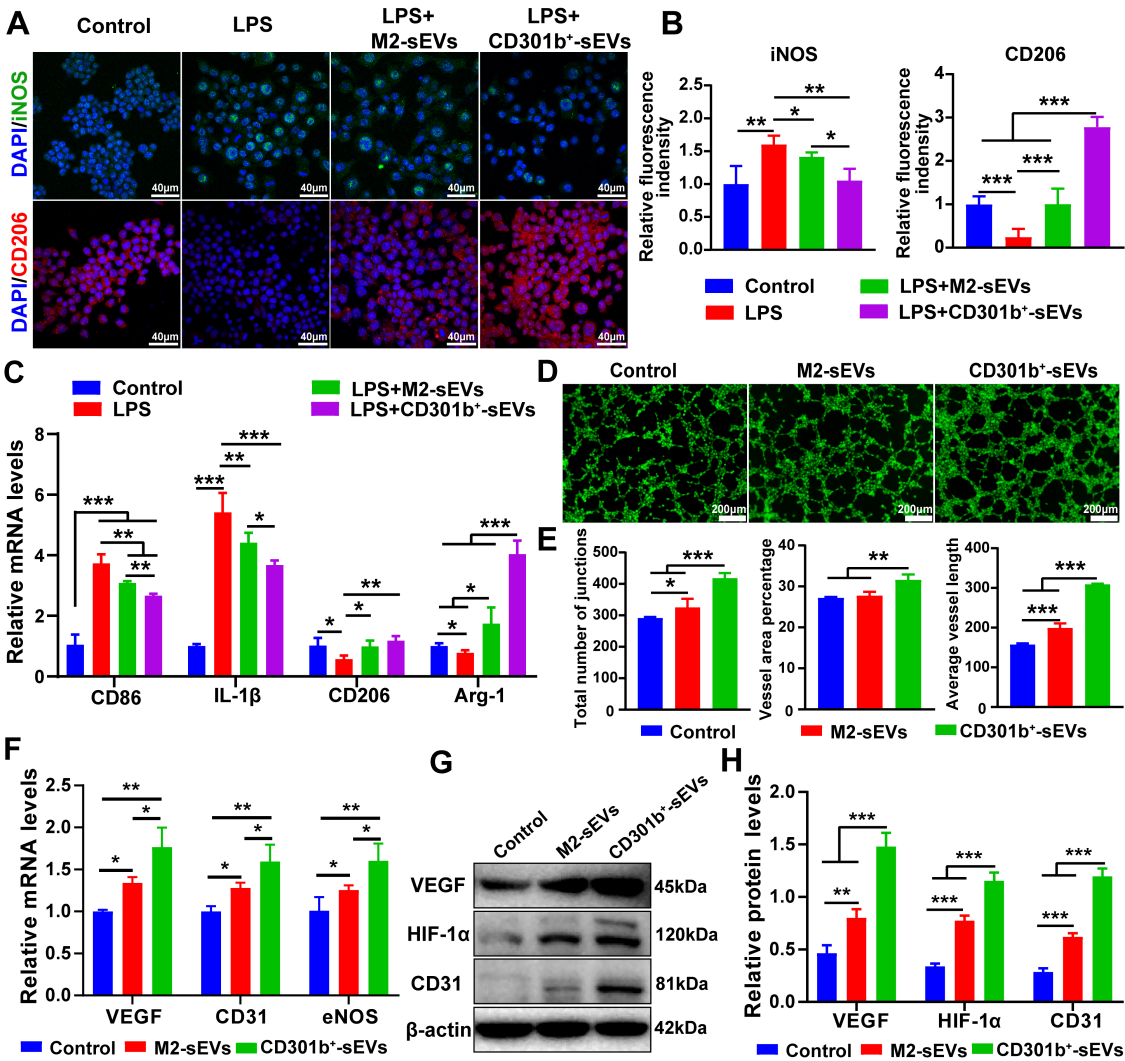
CD301b⁺-sEVs promotes macrophage M2 polarization and angiogenesis *in vitro*. (A) Representative immunofluorescence images showing iNOS (green, M1 marker) and CD206 (red, M2 marker) expression in RAW264.7 cells. Nuclei were stained with DAPI (blue). (B) Quantitative analysis of iNOS and CD206 fluorescence intensity in RAW264.7 cells from panel A, normalized to control group (cells cultured in normal medium without LPS stimulation). (C) RT-qPCR analysis of M1-related genes (CD86, IL-1β) and M2-related genes (CD206, Arg-1) in RAW264.7 cells. Gene expression was normalized to GAPDH and is shown relative to the control group. (D) Representative fluorescent images of HUVECs tube formation on Matrigel. (E) Quantitative analysis of vascular network parameters. (F) RT-qPCR analysis of angiogenic genes expression (VEGF, CD31, and eNOS) in HUVECs. Gene expression was normalized to GAPDH and presented as fold change over the control group (cells cultured in complete medium without added sEVs). (G) Western blot analysis of VEGF, HIF-1α, and CD31 protein expression in HUVECs. (H) Densitometric quantification of protein bands from panel (G), normalized to β-actin.

We next evaluated the immunomodulatory potential of CD301b⁺-sEVs using LPS-activated RAW264.7 cells to mimic the pro-inflammatory bone injury microenvironment. Immunofluorescence analysis demonstrated that CD301b⁺-sEVs exhibits potent dual regulatory capacity: their addition to LPS-activated RAW264.7 cells significantly suppressed the M1 marker iNOS expression while increased the M2 marker CD206 fluorescence intensity compared to the M2-sEVs group** (Figure 2A, B)**, highlighting its superior immunomodulatory properties over conventional M2-sEVs. These observations were confirmed by RT-qPCR analysis. Compared to the LPS group, both sEVs treatments of RAW264.7 cells strongly suppressed the expression of M1-associated genes CD86 and IL-1β, while elevated M2 markers (CD206 and Arg-1). Strikingly, CD301b⁺-sEVs had greater effects than M2-sEVs across all tested markers **(Figure 2C)**. Previous studies have shown that CD301b⁺ macrophages suppress excessive M1-mediated inflammation and promote M2 polarization to support tissue repair 33. In our study, CD301b⁺-sEVs showed a similar immunoregulatory profile. This was in line with the transcriptomic enrichment analysis in **Figure 1** and suggests that CD301b⁺-sEVs may contribute to immune regulation in the bone repair microenvironment.

The angiogenic activity of CD301b⁺-sEVs was then assessed by Matrigel tube formation assay. Results showed that HUVECs treated with CD301b⁺-sEVs formed more complex tubular networks than those treated with M2-sEVs **(Figure 2D)**. Consistent with this observation, the total number of junctions, vessel area percentage, and average vessel length were all increased in the CD301b⁺-sEVs group **(Figure 2E)**, indicating superior pro-angiogenic capability of CD301b^+^-sEVs. This functional superiority was corroborated at the molecular level. RT-qPCR analysis further showed higher mRNA expressions of VEGF, CD31, and eNOS in the CD301b⁺-sEVs group than in the M2-sEVs group **(Figure 2F)**. Western blot results also showed increased expression of VEGF, HIF-1α, and CD31 proteins** (Figure 2G, H)**. These observations are consistent with the GO enrichment results (**Figure 1**) and support the pro-angiogenic activity of CD301b⁺-sEVs. Previous studies reported that CD301b⁺ macrophages, acting as early responders to bioceramic implantation, promote angiogenesis through VEGF upregulation 12. Notably, our results demonstrate that CD301b⁺-sEVs retain the essential functional characteristic of their parent cells.

**Figure 3:**
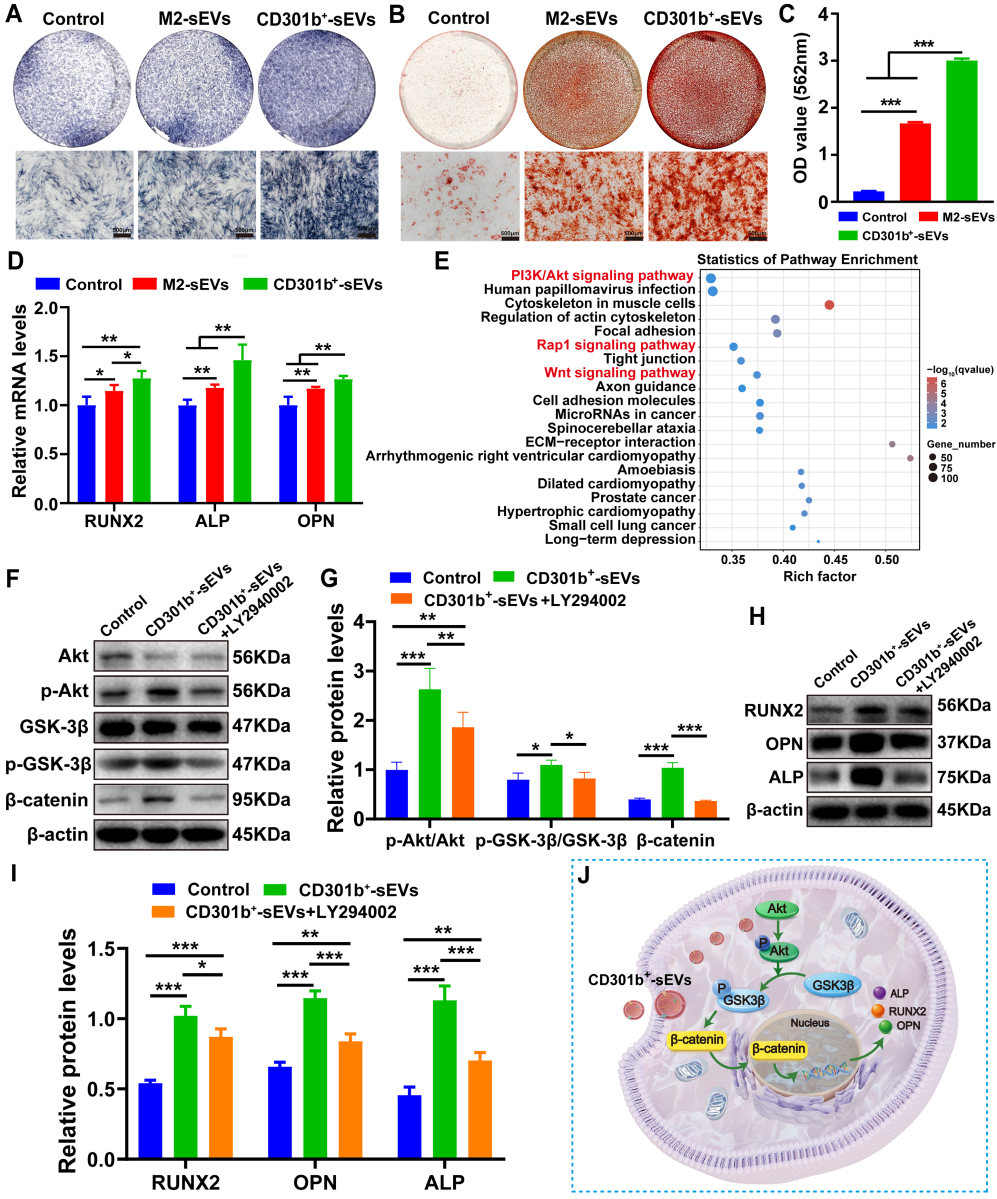
CD301b⁺-sEVs promotes osteogenic differentiation of BMSCs *in vitro*. (A) ALP staining of BMSCs after 6 days of treatments with different types of sEVs. (B) Representative ARS staining of mineralized nodules in BMSCs cultured for 12 days. (C) Quantitative analysis of calcium deposition measured by spectrophotometric absorbance at 562 nm after ARS dissolution. (D) The expression levels of osteogenic-associated genes (RUNX2, ALP, OPN) in BMSCs after 6 days of culture. Data were normalized to GAPDH and expressed as fold change relative to the OIM alone group. (E) KEGG pathway enrichment analysis of DEGs based on the transcriptome sequencing data of CD301b^+^-sEVs. (F) Western blot analysis of Akt/GSK-3β/β-catenin signaling proteins (AKT, p-AKT, GSK-3β, p-GSK-3β, β-catenin) in BMSCs treated with CD301b⁺-sEVs in the presence or absence of the PI3K/Akt inhibitor LY294002 for 6 days. (G) Densitometric quantification of protein bands in (F), normalized to β-actin. (H) Western blot analysis of osteogenic proteins (RUNX2, ALP, OPN) in BMSCs following 6 days of culture under the indicated treatments. (I) Densitometric quantification of protein bands in (H), normalized to β-actin. (J) Proposed mechanism of CD301b⁺-sEVs-mediated osteogenic differentiation via Akt/GSK-3β/β-catenin signaling activation.

We next evaluated the osteogenic regulatory effects of CD301b⁺-sEVs on BMSCs *in vitro* by determining early and late-stage osteogenic differentiation markers. ALP activity, a hallmark of early osteogenic commitment 35, was significantly higher in BMSCs treated with M2-sEVs or CD301b⁺-sEVs than in the OIM-only control group after 6 days of culture **(Figure 3A)**. The increase was more evident in the CD301b⁺-sEVs group than in the M2-sEVs group, as shown by stronger ALP staining. Consistent with this result, ARS staining on day 12 showed more mineralized nodules in the sEVs-treated groups, especially in the CD301b⁺-sEVs group **(Figure 3B)**. Quantification of mineralization further confirmed this pattern **(Figure 3C)**, suggesting that CD301b⁺-sEVs promoted pronounced extracellular matrix calcification, a critical biomarker of osteogenic maturation. At day 6, BMSCs receiving CD301b⁺-sEVs had significantly higher transcript levels of the osteogenic markers RUNX2 (master transcription factor), ALP (early marker), and OPN (late mineralization marker) compared with the other treatment groups **(Figure 3D)**. These findings strongly align with the GO enrichment analysis (**Figure 1**), confirming the robust pro-osteogenic capacity of CD301b^+^-sEVs. Importantly, CD301b⁺-sEVs retained the osteogenic-promoting properties of their parent cells, consistent with reports that CD301b⁺ macrophage supernatant induces BMSCs osteogenesis 11, 33. However, this functional inheritance is not unique to macrophage-derived sEVs. For instance, sEVs from BMSCs carry osteogenesis-related miRNAs and proteins, effectively replicating the pro-regenerative functions of their parent cells 36. Similarly, sEVs sourced from adipose-derived mesenchymal stem cells (ADSCs) inherit the immunomodulatory and tissue repair properties of parental ADSCs 37. Although many existing sEVs-based bone regeneration strategies also exert combined immunomodulatory, pro-angiogenic, and pro-osteogenic effects through their parental cell-derived cargos, CD301b⁺-sEVs are distinguished by their derivation from a repair-associated CD301b⁺ macrophage subset. In particular, CD301b⁺-sEVs exhibited superior anti-inflammatory, angiogenic, and osteogenic activities compared with M2-sEVs, suggesting their advantage in coordinating the immune-vascular-osteogenic process during bone repair.

To better understand how CD301b⁺-sEVs promote osteogenesis, we conducted KEGG pathway enrichment analysis based on the transcriptome sequencing data. As shown in **Figure 1E**, significant enrichment of DEGs was observed in the PI3K/Akt, Rap1, and Wnt signaling pathways. These pathways are recognized to contribute to osteogenic differentiation and angiogenesis during bone repair 38, 39. Among them, PI3K/Akt showed the strongest enrichment in our analysis. Moreover, this pathway is also known to be important in MSC osteogenic differentiation 40. Mechanistically, the PI3K/Akt pathway can act as a central regulatory node that crosstalks with Wnt signaling. Activation of PI3K/Akt promotes GSK-3β phosphorylation on serine 9, which suppresses GSK-3β function and reduces β-catenin degradation. Stabilized β-catenin can then accumulate in the cytoplasm and translocate into the nucleus, where it promotes transcription of genes involved in bone formation, including RUNX2 and ALP 41. Therefore, we focused on the PI3K/Akt pathway for subsequent mechanistic validation. Accordingly, we next examined whether CD301b^+^-sEVs might regulate the osteogenesis of BMSCs through the Akt/GSK-3β/β-catenin signaling cascade. Western blot results revealed that CD301b⁺-sEVs treatment for 6 days significantly increased the p-Akt/Akt ratio, the p-GSK3β/GSK3β ratio, and the β-catenin protein levels relative to the control group **(Figure 3F, G)**. Correspondingly, these molecular changes were accompanied by the upregulation of osteogenic marker proteins (RUNX2, ALP, and OPN)** (Figure 3H, I)**, indicating enhanced osteogenic potential of BMSCs. In contrast, pharmacological inhibition of PI3K/Akt signal transduction axis with LY294002, the phosphorylation of Akt and GSK3β by CD301b⁺-sEVs was significantly inhibited, leading to reduced β-catenin accumulation and a concomitant decrease in the expression of osteogenic proteins** (Figure 3F-I)**. Collectively, these results support that CD301b^+^-sEVs promotes osteogenesis by activating the Akt/GSK-3β/β-catenin axis **(Figure 3J)**, although the potential involvement of Rap1 and other Wnt-related components warrants further investigation in the future.

Although sEVs derived from MSCs, osteoblasts, endothelial cells, or M2 macrophages have been reported to promote bone regeneration, few of these sEVs populations have been shown to robustly integrate all three processes-immunomodulation, angiogenesis, and osteogenesis-within a single platform 42. Moreover, emerging evidence indicates that specific cell subpopulations can produce sEVs with superior regenerative potency compared to their parental or bulk cell populations. For instance, sEVs derived from CD271⁺CD56⁺ BMSC subpopulations promoted axon regeneration and neurological recovery more effectively than conventional BMSC sEVs in spinal cord injury models 29. Similarly, CD133⁺ urine-derived stem cell sEVs outperformed total urine-derived stem cell sEVs in promoting chondrogenic differentiation and rotator cuff tendon-bone healing 43. By leveraging CD301b⁺ macrophages, a recently recognized pro-regenerative subset that reportedly outperforms conventionally M2 macrophages in tissue repair, we demonstrate for the first time that CD301b⁺-sEVs exhibit markedly stronger anti-inflammatory, pro-angiogenic, and pro-osteogenic activities than M2-sEVs. Thus, CD301b⁺-sEVs provide a functionally superior cell-free platform for coordinating the immune-vascular-osteogenic cascade in bone regeneration.

### Preparation and characterization of microspheres

**Figure 4:**
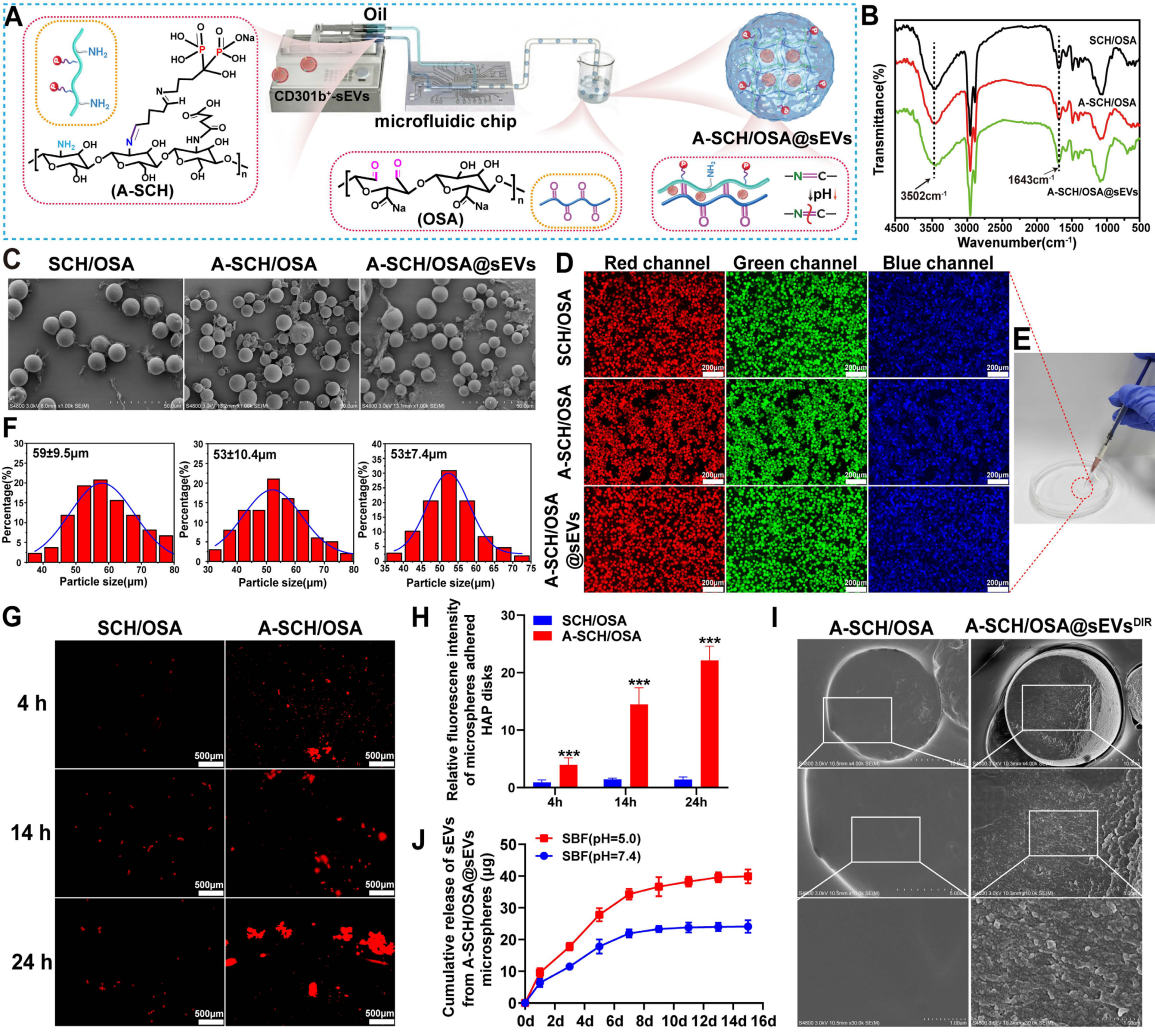
Synthesis and characterization of microspheres. (A) Schematic flowchart of microsphere preparation using a microfluidic chip. FT-IR spectra (B) and representative SEM morphology (C) of the various microspheres. (D) Fluorescence imaging under standard DAPI, FITC, and TRITC filter sets. (E) Injectability of microspheres through a 32 G needle. (F) Particle size distribution of microspheres. Fluorescence imaging (G) and quantitative fluorescence intensity (H) of microspheres adhered to HAP disks. (I) SEM morphology of cryosections from A-SCH/OSA@sEVs^DIR^ microsphere. (J) CD301b^+^-sEVs release profiles of A-SCH/OSA@sEVs microspheres in either acidic (pH 5.0) or neutral (pH 7.4) simulated fluids.

Despite their therapeutic promise, the direct injection of sEVs into bone defect sites faces challenges of limited retention and susceptibility to rapid clearance 44. To overcome these limitations, we fabricated an injectable, multifunctional microsphere carrier using microfluidics for sustained sEVs release. Specifically, microfluidic flow-focusing chips (featuring two inlets and one outlet) were employed to fabricate microspheres via a two-step method. In the first stage, aqueous phases (SCH, A-SCH or A-SCH containing CD301b^+^-sEVs) and oil phases (mineral oil with 5% v/v Span80) converged at flow-focusing junctions to generate monodisperse droplets. During the second stage, these droplets were dispersed in the oil phase and collected in an OSA solution. Crosslinking via Schiff base bonds between amino groups of SCH and aldehyde moieties of OSA yielded stable hydrogel microspheres (SCH/OSA, A-SCH/OSA, and A-SCH/OSA@sEVs) **(Figure 4A, Figure S7A)**. Structural validation was performed using FT-IR and ^31^P NMR spectroscopy. The FT-IR spectrum of OSA exhibited a weak aldehyde peak at 1725 cm⁻¹ **(Figure S7B)**, which was absent in the spectrum of SA, confirming the successful oxidation of the hydroxyl groups to aldehyde functionalities 45. SCH derivatives showed characteristic amide I and amide II absorption peaks at 1647 cm⁻¹ and 1554 cm⁻¹, respectively, indicating successful acylation. In A-SCH, an additional N-H stretching peak was observed at 3502 cm⁻¹ **(Figure S7C)**, consistent with amide bond formation 46. In all microsphere groups, a clear peak representing the imine bond (-C=N-) appeared at 1643 cm⁻¹ **(Figure 4B)**, suggesting the formation of Schiff base crosslinking 25. In addition, ^31^P NMR analysis showed a resonance peak at 17.6 ppm 28 in both ALE and A-SCH **(Figure S7D)**, supporting conjugation of ALE to SCH. These data together support the successful synthesis of the modified materials.

The morphological features and size distribution of the microspheres were then analyzed. SEM showed that all microspheres had smooth surfaces and relatively uniform sizes **(Figure 4C).** Similar results were obtained by inverted fluorescence microscopy, which showed a regular spherical shape and detectable autofluorescence. Under DAPI, FITC, and TRITC filter sets, the microspheres emitted blue, green, and red fluorescence, respectively **(Figure 4D)**, probably because of the intrinsic fluorescence of CH 46. The average diameters of the microspheres were measured as 59 ± 9.5 μm (SCH/OSA), 53 ± 10.4 μm (A-SCH/OSA), and 53 ± 7.4 μm (A-SCH/OSA@sEVs), respectively **(Figure 4F)**. The microspheres were also injectable via a 32 G needle (inner diameter, 110 μm) without obvious clogging and remained intact after injection **(Figure 4E)**. These results suggest that the microspheres are suitable for use as injectable implants.

ALE is an FDA-approved bisphosphonate with strong binding ability for hydroxyapatite (HAP), the major mineral constituent of bone, through binding to calcium ions. This property is retained after conjugation to carrier molecules 47. We therefore examined the bone-targeting ability of ALE-modified microspheres (A-SCH/OSA)* in vitro* and* in vivo*. *In vitro*, HAP disks were used to mimic the mineral phase of bone and to assess the binding of A-SCH/OSA microspheres 48. After incubation and washing, fluorescence imaging showed greater retention of A-SCH/OSA microspheres on HAP disks than SCH/OSA microspheres at 4, 14, and 24 h **(Figure 4G)**. The quantitative results showed the same trend, with significantly higher binding in the A-SCH/OSA group at each time point **(Figure 4H)**. This difference is likely related to the interaction between ALE and calcium ions in HAP 27. For *in vivo* evaluation, Cy5.5-labeled microspheres were tested using a rat model of calvarial defect. Real-time fluorescence imaging **(Figure S8A)** revealed that A-SCH/OSA^Cy5.5^ microspheres remained at the defect site longer than SCH/OSA^Cy5.5^ microspheres, further supported by quantitative analysis indicating a substantially slower signal decay rate **(Figure S8B)**. These results indicate that ALE modification achieved defect-specific and improved microsphere retention at the bone defect site and may support sustained local delivery of CD301b⁺-sEVs.

To investigate sEVs encapsulation within microspheres, DIR-labeled CD301b⁺-sEVs (sEVsᴰᴵᴿ) were loaded into microspheres to prepare A-SCH/OSA@sEVsᴰᴵᴿ. SEM image of microsphere cross-sections showed a rougher internal surface with granular structures in A-SCH/OSA@sEVsᴰᴵᴿ, while the A-SCH/OSA group showed a relatively smooth surface** (Figure 4I)**. CLSM further revealed a uniform DIR fluorescence signal within the cross-sections of A-SCH/OSA@sEVs^DIR^ under near-infrared excitation, while no signal was observed in A-SCH/OSA controls** (Figure S9)**. These observations support successful loading of CD301b⁺-sEVs into the microspheres.

Because bone defects are often accompanied by hypoxia and persistent inflammation, the local extracellular environment tends to become acidic 49, 50. Based on this feature, we evaluated the pH-dependent release of CD301b⁺-sEVs from A-SCH/OSA@sEVs microspheres. As shown in **Figure 4J**, under acidic conditions (SBF, pH 5.0), CD301b⁺-sEVs release was faster than that observed at physiological pH (7.4). The release increased rapidly in the initial 7 days, then entered a slower phase and approached a plateau by day 13. The cumulative release reached 39.62 ± 1.58 μg at day 13, compared with 9.54 ± 1.43 μg on day 1. By contrast, release at pH 7.4 was more gradually and remained at a lower level throughout the observation period. No obvious burst release was observed over 15 days, suggesting that the sEVs were stably incorporated into the A-SCH/OSA network and released in a controlled manner. SEM examination on day 15 showed clear structural differences between the two pH conditions **(Figure S10)**. Microspheres incubated at pH 5.0 showed partial shrinkage, collapse, and deformation, whereas those maintained at pH 7.4 largely preserved their spherical structure, with only slight surface changes. This difference may be related to the dynamic Schiff base bonds in the network, which are more susceptible to hydrolysis under acidic conditions 26. The faster release under acidic conditions may be advantageous for early local release in the inflammatory niche, while the sustained release pattern may help maintain the effect during later stages of bone repair. Therefore, A-SCH/OSA@sEVs microsphere system is specifically designed to leverage the pathological acidity of bone defects to achieve spatiotemporal control over sEVs delivery, aligning release patterns with the biological timeline of bone repair.

### Multiple effects of A-SCH/OSA@sEVs microspheres *in vitro*

#### Evaluation of the immunomodulatory capability of microspheres *in vitro*

**Figure 5:**
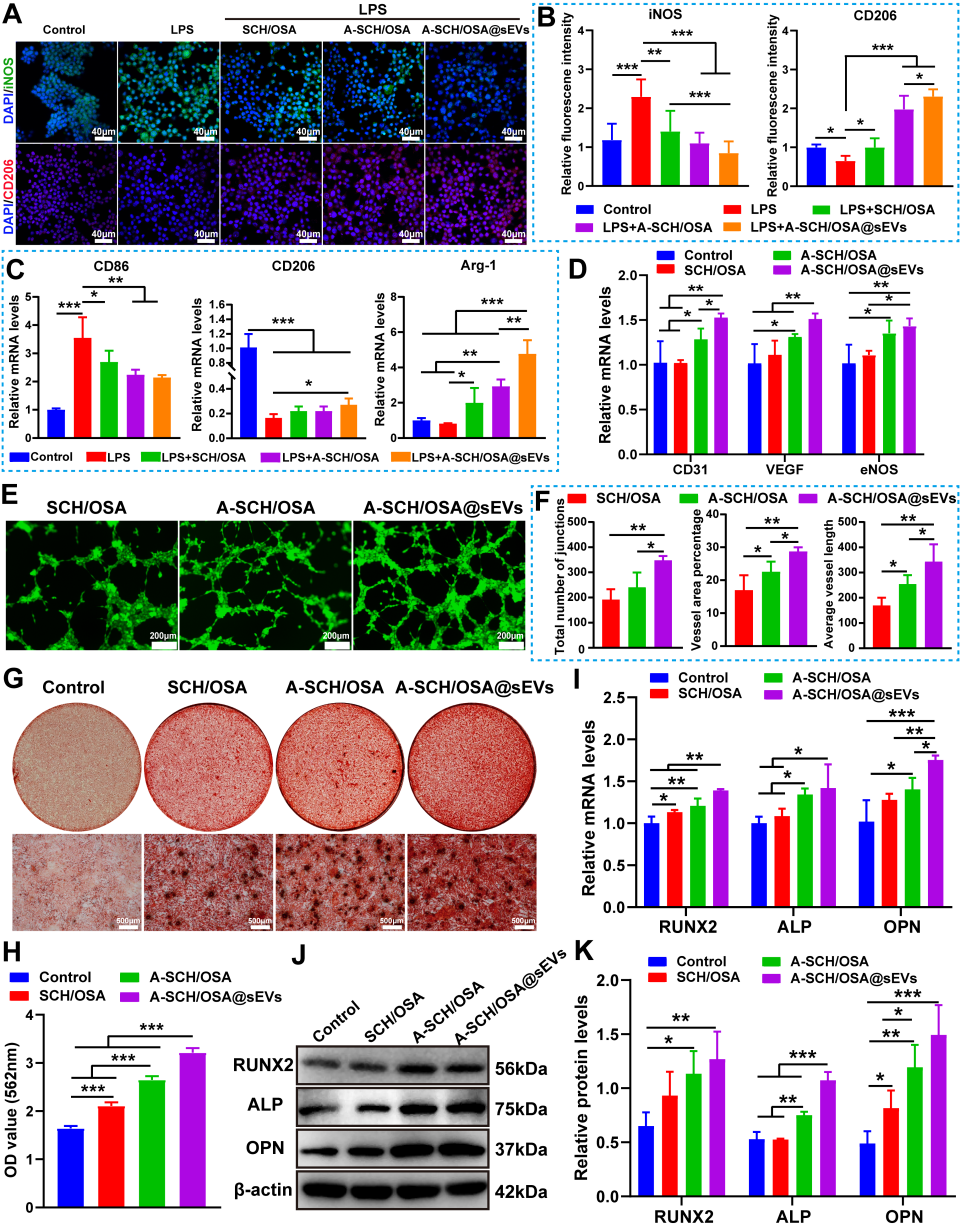
Verification of the potential of A-SCH/OSA@sEVs microspheres in promoting macrophage polarization, angiogenesis, and osteogenic differentiation* in vitro*. (A) The expression of iNOS (green) and CD206 (red) in RAW264.7 cells treated with different types of microspheres; nuclei were labeled with DAPI (blue). (B) Quantitative analysis of iNOS and CD206 expressions based on fluorescence density, normalized to the control group (cells cultured in normal medium without LPS stimulation). (C) RT-qPCR analysis of CD86, CD206 and Arg-1 gene expression in RAW264.7 cells treated with different microspheres. (D) RT-qPCR analysis of angiogenic gene expression (VEGF, CD31, and eNOS) in HUVECs treated with various microspheres. The results were normalized to GAPDH and shown as relative expression compared with the control group (cells cultured in complete medium without added sEVs). (E) Fluorescence images showing tube formation by HUVECs. (F) Quantitative analysis of vascular network parameters. (G) ARS staining of calcium deposits in BMSCs treated with various microspheres for 12 days. (H) Quantitative analysis of calcium deposition (absorbance at 562 nm after ARS dissolution). (I) Gene expression of ALP, RUNX2, and OPN in BMSCs after 6 days of culture. The relative expression (normalized to GAPDH) is shown compared with OIM alone (control). (J) Protein expression of RUNX2, ALP, and OPN in BMSCs after 6 days of treatment. (K) Densitometric quantification of protein bands in (J), normalized to β-actin [SCH/OSA: plain microsphere fabricated by composition of SCH and OSA; A-SCH/OSA: ALE-modified bone-targeting microsphere composed of A-SCH and OSA; A-SCH/OSA@sEVs: A-SCH/OSA microsphere encapsulated with CD301b^+^-sEVs].

To evaluate immunomodulatory effects of the microspheres, RAW264.7 cells were treated with LPS to simulate inflammation and drive M1 polarization, while cells cultured in normal medium without LPS stimulation served as control. Immunofluorescence results **(Figure 5A)** revealed that LPS significantly upregulated the level of the M1-associated marker iNOS. In contrast, after adding different microspheres (SCH/OSA, A-SCH/OSA, or A-SCH/OSA@sEVs) to the apical side of Transwell inserts positioned above the cells, iNOS expression was suppressed, with the most prominent suppression observed in the A-SCH/OSA@sEVs group. Conversely, LPS minimized CD206 expression, while all microspheres induced a specific perinuclear overexpression of CD206 in RAW264.7 cells, with the strongest effect again seen in the A-SCH/OSA@sEVs group. Quantitative analysis of the fluorescence density, normalized to the control group, further supported these observations **(Figure 5B)**. Gene expression analysis further confirmed the phenotypic switching **(Figure 5C)**. LPS elevated CD86 expression by 3.55-fold compared to the control group, whereas all microsphere groups significantly downregulated CD86 expression, particularly the A-SCH/OSA@sEVs group (2.15 ± 0.08, *P* < 0.01 *vs.* LPS group). Regarding M2 markers, LPS reduced their expression to 0.16 ± 0.03 (CD206) and 0.82 ± 0.03 (Arg-1). By contrast, exposure of cells to all types of microspheres upregulated both markers. Notably, the A-SCH/OSA@sEVs group displayed the strongest M2-polarizing capacity, with CD206 and Arg-1 expression reaching 0.27 ± 0.05 (*P* < 0.05 *vs.* LPS group) and 4.77 ± 1.00 (*P* < 0.001 *vs.* LPS group), respectively. These findings indicate that all microspheres promote M1-to-M2 polarization in RAW264.7 cells. This is likely attributable to the immunomodulatory properties of CH, which is found to drive M2 polarization of macrophages by upregulating CD206 and suppressing M1 markers (e.g., HLA-DR, CD86) 51, 52. The enhanced bioactivity of A-SCH/OSA microspheres compared to SCH/OSA may be due to ALE, which reduces inflammatory cell infiltration and suppresses key pro-inflammatory cytokines including IL-1β, IL-6, TNF-α, IFN-γ, and iNOS in inflammatory models 53. However, the superior performance of A-SCH/OSA@sEVs likely is probably due to the prolonged release of CD301b^+^-sEVs, which synergizes with the immune modulatory property of microspheres amplifying M2 polarization.

#### Evaluation of the angiogenesis effects of microspheres *in vitro*

Angiogenesis is essential for bone regeneration, as functional vascular networks ensure oxygen and nutrient supply during bone healing 54. Therefore, we first assessed the angiogenic capacity of the microspheres *in vitro* using HUVECs at both molecular and functional levels. RT-qPCR analysis **(Figure 5D)** revealed that compared to the SCH/OSA group, A-SCH/OSA microspheres mildly upregulated CD31 expression but had no statistically significant impact on VEGF or eNOS. In contrast, the key angiogenic factors such as CD31 and VEGF were strongly enhanced in SCH/OSA@sEVs group, when in comparison to all other groups. Although eNOS expression also showed an increasing trend, the difference was not statistically significant compared with the A-SCH/OSA group. eNOS primarily functions to catalyze the production of nitric oxide, which is mainly involved in the maturation and functional stabilization of newly formed vessels 55. Its expression generally peak typically occurs later than that of early markers such as VEGF and CD31.

Then we further evaluated angiogenesis using a Matrigel-based* in vitro* assay. Results demonstrated **(Figure 5E, F)** that as compared with other groups, the A-SCH/OSA@sEVs group exhibited the most potent pro-angiogenic performance, as evidenced by densely branched tubular networks, significantly higher numbers of junctions, increased vessel area percentage, and longer average vessel length. These results suggest that sustained release of CD301b⁺-sEVs improves the pro-angiogenic effect of A-SCH/OSA microspheres.

#### Evaluation of osteogenic effects of microspheres *in vitro*

Because the osteoinductive capacity of biomaterials is critical for repairing localized bone defects 56. We then assessed how the microspheres affect BMSCs osteogenic differentiation. Pre-adherent BMSCs were cultured in OIM alone (control) or in OIM containing microspheres, and osteogenesis was evaluated by ARS staining, RT-qPCR, and western blotting.

After 12 days of co-culture, ARS results **(Figure 5G)** demonstrated more mineralized nodules in every microsphere-treated group relative to the control. The extent of mineralization improved in the order of SCH/OSA, A-SCH/OSA, and A-SCH/OSA@sEVs. Among these groups, A-SCH/OSA@sEVs showed the strongest ARS staining, with heavy calcium deposition surrounding the microspheres. The quantitative data **(Figure 5H)** agreed with the staining results and indicated that the A-SCH/OSA@sEVs group had the highest absorbance value among all groups.

Similarly, RT-qPCR data showed that the transcript levels of RUNX2, ALP, and OPN mRNA were elevated in all microsphere-treated groups **(Figure 5I)**. The greatest expression was seen in the A-SCH/OSA@sEVs group, with all three markers elevated relative to both the control and the A-SCH/OSA group. Western blot analysis **(Figure 5J, K)** further confirmed that the protein expression trends across groups were highly consistent with the gene levels. In particular, RUNX2, ALP, and OPN were all more abundant in the A-SCH/OSA@sEVs group relative to the other groups. These multi-modal data conclusively demonstrate the superior osteoinductive capacity of A-SCH/OSA@sEVs microspheres, which arises from a synergistic combination of mechanisms. First, CH itself possesses inherent osteoinductive properties, which has been shown to facilitate osteogenesis of BMSCs by increasing the expression of osteogenesis-associated genes and calcium-binding proteins 57. Second, the incorporation of ALE within the microsphere’s matrix further enhances osteoinduction. Both free and carrier-bound ALE have been shown to enhance MSC osteogenesis by increasing osteogenic markers levels, including OPN, ALP, OCN, and laminin 58, 59. Consistently with these reports, the A-SCH/OSA group in this study displayed greater osteogenic activity than the SCH/OSA group, confirming that ALE retains its bioactivity within the microsphere system. In addition, sustained release of CD301b⁺-sEVs may further enhance the activation of osteogenesis-related signaling pathways. This multi-mechanistic synergy endows A-SCH/OSA@sEVs with remarkable bone regeneration efficacy, offering an innovative strategy for bone defect repair.

### Calvarial bone defect regeneration

**Figure 6:**
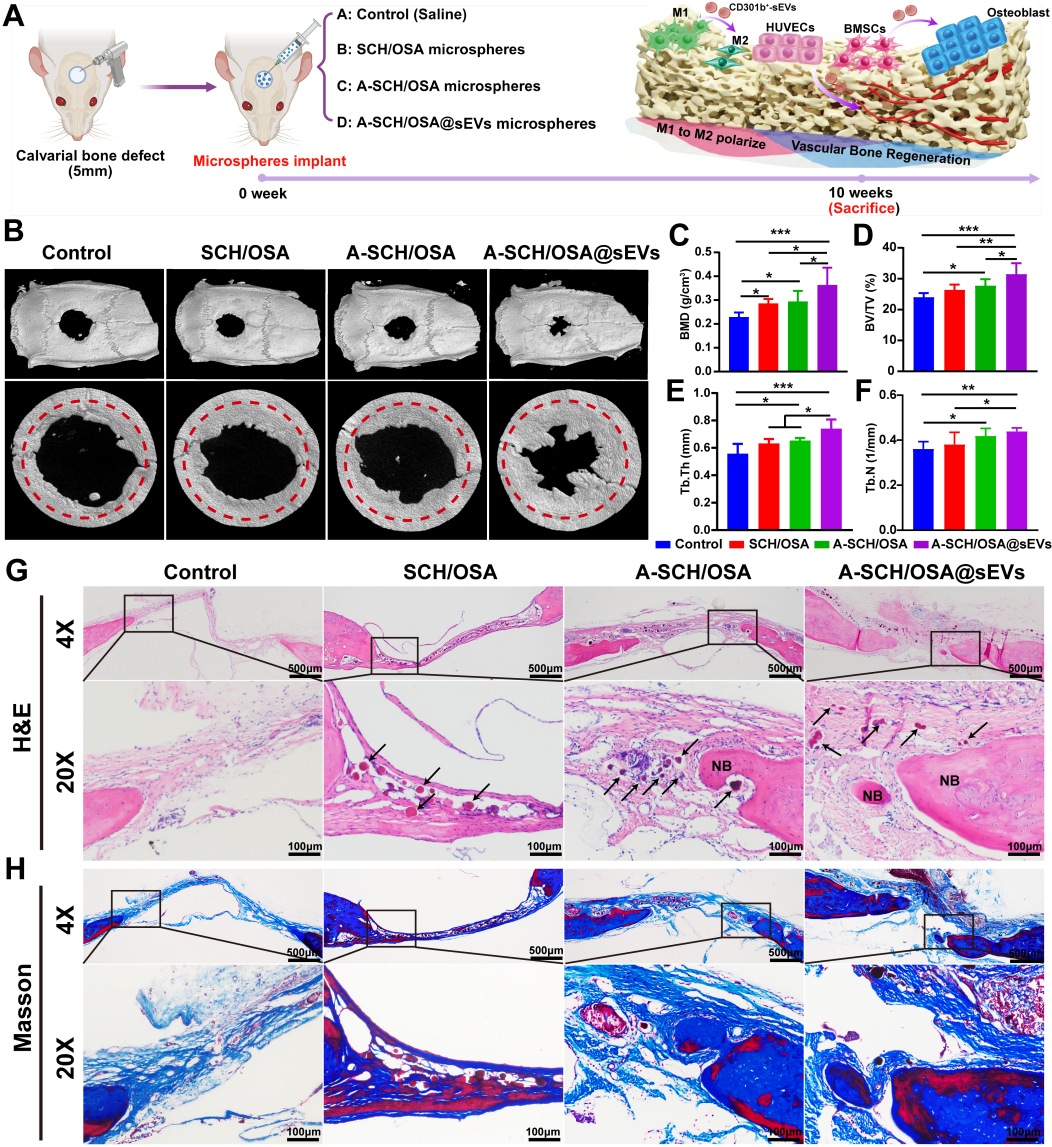
A-SCH/OSA@sEVs microspheres promote calvarial bone regeneration. (A) Schematic illustrating the calvarial bone defect model and surgical implantation procedure. (B) Reconstructed micro-CT images (both 3D and 2D) of skull defects captured 10 weeks post-surgery (n = 5). Red circles show areas of new bone generation. Quantitative analysis of BMD (C), BV/TV (D), Tb.Th (E), and Tb.N (F) in defects filled with various microspheres. Representative H&E staining (G) and Masson’s trichrome-stained sections (H) of calvarial defects after microsphere implantation. Black arrowheads indicate the residual, degrading microsphere fragments, and NB denotes newly formed bone. [The microspheres are identical to those described in **Figure 5;** the control (defect-only) group injected with the same volume of saline].

Based on the immunomodulatory, angiogenic, and osteogenic regulatory capacities of A-SCH/OSA@sEVs microspheres demonstrated* in vitro*, we further systematically evaluated their bone repair efficacy in a rat calvarial defect setting. As illustrated in **Figure 6A**, 5-mm circular defects were created along the skull midline, into which SCH/OSA, A-SCH/OSA, or A-SCH/OSA@sEVs microspheres were implanted. The control animals were treated with an equal volume of saline alone. Skull specimens were collected at 10 weeks post-surgery for bone regeneration assessment.

As seen from the 3D Micro-CT images** (Figure 6B),** the defect-only control group exhibited only minimal new bone formation, with persistent central defects, indicating a limited innate regenerative capacity. Conversely, each microsphere-treated group exhibited bone repair, albeit to different degrees. Compared with the control, the SCH/OSA group showed a clear increase in BMD, whereas BV/TV, Tb.Th, and Tb.N remained largely unchanged. The A-SCH/OSA group showed a tendency toward enhanced bone repair than the SCH/OSA group, but the differences did not reach statistical significance. In contrast, loading CD301b⁺-sEVs further improved the regenerative effect. Quantitative analysis **(Figure 6C-F)** showed that the A-SCH/OSA@sEVs group exhibited higher BMD, BV/TV, and Tb.Th than the A-SCH/OSA group. Specifically, BMD reached to 0.36 ± 0.07 g/cm³ (23.54% higher), BV/TV to 31.46 ± 3.54% (13.49% higher), and Tb.Th to 0.74 ± 0.07 mm (13.17% higher). Although Tb.N (0.44 ± 0.02 1/mm) showed an increasing trend, the difference was not statistically significant.

Histological evaluation was further performed to assess bone regeneration in the defect area. As revealed by H&E staining **(Figure 6G),** in the control group, which received no microsphere implantation, the defect was mainly filled with a thin layer of fibrous tissue with only a few osteoblasts, and little signs of new bone formation or neovascularization. In the SCH/OSA group, fibrous tissue was denser, but bone and vessel formation was still minimal. A few microspheres (black arrows) appeared in the defect area and showed partial degradation. By comparison, both the A-SCH/OSA and A-SCH/OSA@sEVs groups showed more extensive and continuous new bone formation, together with more obvious bone matrix deposition. In these two groups, the microspheres appeared more degraded, with fragmented structures and closer contact with the surrounding tissue. Particularly, in the A-SCH/OSA@sEVs group, the remaining microspheres were highly fragmented and embedded within a denser bone matrix. These fragments were infiltrated by host cells and closely associated with newly formed bone, suggesting good biodegradation and tissue integration. The accelerated degradation and improved bone integration may be related to greater cellular infiltration and the active participation of osteogenic cells and M2-polarized immune cells, which can secrete matrix-remodeling enzymes 60 and promote bio-resorption of the microspheres.

Masson’s trichrome staining** (Figure 6H)** showed a similar pattern. Only a few blue-stained collagen fibers and very little new bone were observed in the control group. In contrast, all microsphere-treated groups showed increased collagen fiber deposition. Among them, the A-SCH/OSA@sEVs group exhibited the thickest and most densely arranged collagen fiber layers, indicating a stronger osteogenic response. These histological observations align with the micro-CT results and further support the superior bone repair effect of A-SCH/OSA@sEVs microspheres.

To evaluate the systemic biosafety of the microsphere, sections of the main organs (including heart, liver, spleen, kidneys, and lungs) were examined 61. As shown in **Figure S11**, no obvious pathological changes or marked inflammatory cell infiltration were found in any group. This result suggests that local implantation of the microspheres did not produce detectable systemic toxicity during degradation. *In vitro* cytocompatibility was further assessed by live/dead staining, CCK-8 assays, and cytoskeletal staining. All microsphere formulations showed good cytocompatibility, with high cell viability and normal cell spreading (**Figure S12**). The combination of controlled local degradation and absence of systemic toxicity underscores the excellent biocompatibility and high therapeutic potential of the developed microsphere systems.

**Figure 7:**
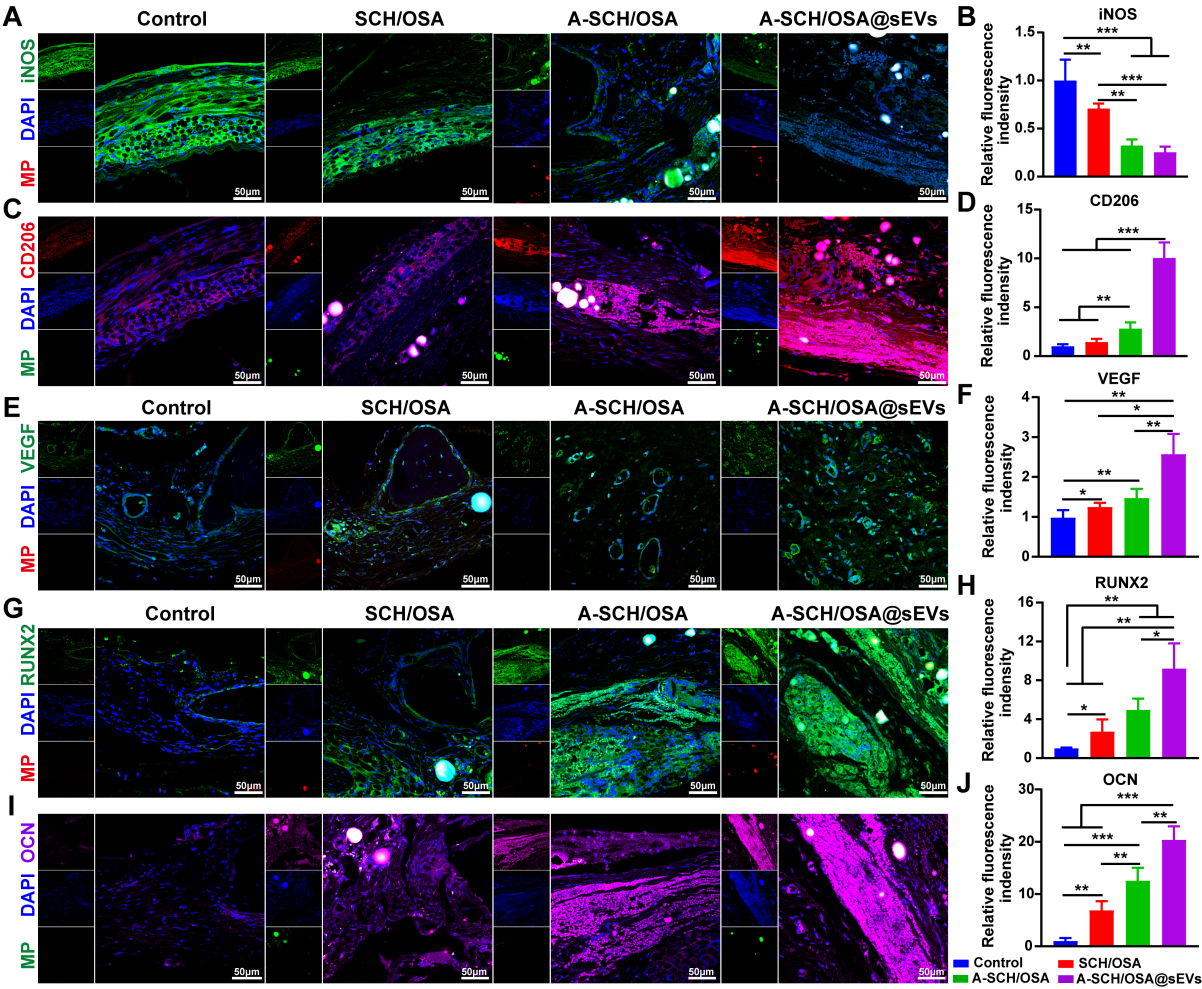
Analysis of macrophage polarization, angiogenesis, and osteogenesis of A-SCH/OSA@sEVs microspheres (abbreviated as MP) *in vivo*. Representative immunohistochemical fluorescence images of the M1 marker iNOS (green, A) and M2 marker CD206 (red, C) at the defect site; nuclei were counterstained with DAPI (blue). Quantitative analysis of iNOS (B) and CD206 (D) expression levels based on fluorescence density. Representative immunohistochemical fluorescence images showing the vascular endothelial markers VEGF (E). Quantitative analysis of the fluorescence intensity of VEGF (F). Representative immunohistochemical fluorescence images of the osteogenic markers RUNX2 (G) and OCN (I). Quantitative analysis of the fluorescence intensity for the respective osteogenic markers RUNX2 (H) and OCN (J). [The microspheres are the same as described in **Figure 5;** the control (defect-only) group received a saline injection].

Macrophage polarization during the early inflammatory phase critically regulates the local bone regenerative microenvironment, where the transition from the pro-inflammatory M1 state to the pro-healing M2 phenotype promotes angiogenesis and osteogenic repair 62. Accordingly, macrophage polarization within the defect area was investigated *in vivo*. Immunohistochemical fluorescence staining for iNOS, CD86 (M1-markers) and CD206 (M2-marker) were performed **(Figure 7A-D and Figure S13)**. In the control group, abundant iNOS- and CD86-positive signals with minimal CD206 staining were observed in the defect zone. In contrast, the microsphere-treated groups exhibited an M2 polarization trend, with CD206-positive staining increasing in the order of SCH/OSA < A-SCH/OSA < A-SCH/OSA@sEVs, accompanied by reduced iNOS and CD86 signals. The A-SCH/OSA@sEVs group showed the strongest M2 polarization among all groups, consistent with the quantitative results **(Figure 7B, D and Figure S13B)**. To further examine cytokine changes associated with macrophage polarization, immunohistochemical fluorescence analysis was conducted to assess TNF-α and IL-10 levels. As shown in **Figure S14**, strong TNF-α staining and weak IL-10 expression were observed in the control group, indicating a persistent pro-inflammatory environment in the defect area. In the SCH/OSA and A-SCH/OSA groups, TNF-α expression was reduced and IL-10 expression was moderately increased compared with the control group, indicating partial alleviation of local inflammation. Notably, the A-SCH/OSA@sEVs group exhibited the lowest TNF-α expression and the highest IL-10 expression among all groups, consistent with the quantitative analysis **(Figure S14B-C)**. Given that TNF-α is a typical pro-inflammatory cytokine linked to M1 macrophage activation, whereas IL-10 is an anti-inflammatory cytokine characteristic of the M2 phenotype 63, these findings are consistent with the reduced iNOS/CD86 and increased CD206 expression observed above. Collectively, these findings suggest that A-SCH/OSA@sEVs attenuates the local inflammatory response and helps create a pro-regenerative immune milieu.

Bone regeneration requires a functional vascular network to provide oxygen and nutrients, and the coordinated coupling of angiogenesis and osteogenesis plays a central role in orchestrating the staged progression of bone repair 64. To evaluate angiogenesis *in vivo*, immunohistochemical fluorescence staining for VEGF, CD31 and α-SMA was performed **(Figure 7E-F and Figure S15).** The control group exhibited sparse vascular structures with weak fluorescence signals in the defect area. By comparison, A-SCH/OSA@sEVs groups exhibited higher density of VEGF-positive signals and CD31⁺/α-SMA⁺ co-localized microvessels compared with the other groups, indicating improved vascular formation and maturation. Quantitative analysis showed the same trend** (Figure 7F and Figure S15B, C)**. These findings demonstrate that A-SCH/OSA@sEVs not only promotes angiogenesis but also enhances vascular maturation, supporting the formation of more mature vascular structures in the regenerated tissue. In terms of osteogenic potent, immunohistochemical fluorescence staining of ALP, RUNX2, and OCN (marker of late osteogenic differentiation) **(Figure 7G-J and Figure S16)** demonstrated that the control group exhibited weak fluorescence signals, whereas the microsphere-treated groups showed stronger osteogenic marker expression, increasing in the order of SCH/OSA < A-SCH/OSA < A-SCH/OSA@sEVs. Notably, the A-SCH/OSA@sEVs group displayed the most robust staining intensity, consistent with the quantitative analysis** (Figure 7H, J and Figure S16B)**.

Collectively, these findings indicate that incorporation of CD301b⁺-sEVs significantly enhances the ability of A-SCH/OSA microspheres to promote M2 macrophage polarization during the inflammatory stage of bone healing, thereby establishing a pro-regenerative immune microenvironment. It has been reported that M2-polarized macrophages facilitate angiogenesis and osteogenic differentiation through the secretion of anti-inflammatory cytokines, pro-angiogenic mediators such as VEGF and pro-osteogenic mediators such as BMP2 65. VEGF/VEGFR2 signaling may activate downstream MAPK/ERK and PI3K-AKT pathways to support endothelial activation and angiogenesis 66. In parallel, BMP/BMPR signaling may activate the SMAD1/5/8–SMAD4 axis to promote osteoblast-specific gene expression and osteogenic differentiation 67. Enhanced vascular formation, as observed in the A-SCH/OSA@sEVs group, provides essential oxygen, nutrients, and angiocrine signals to support osteogenic differentiation and matrix mineralization 68. Conversely, newly formed osteogenic cells can secrete pro-angiogenic factors (e.g., VEGF, PDGF) that stimulate endothelial cell proliferation and migration and further support the coupling between angiogenesis and osteogenesis 64, 69. Pathways such as PDGF/PDGFRβ, HIF-1α/VEGF and Notch may also contribute to this bidirectional crosstalk 64. These results suggest that A-SCH/OSA@sEVs microspheres promote bone regeneration by improving the coordination among immune regulation, vascular formation, and osteogenesis. This integrated mechanism aligns with recent advances in regenerative medicine, where multidimensional modulation of the local microenvironment has become increasingly emphasized. For instance, multifunctional platforms based on metal-polyphenol networks 70 or supramolecular hydrogels 71 have been reported to concurrently modulate inflammation, vascularization, and tissue formation, thereby improving repair outcomes.

Although this study demonstrates that the bone-targeting and stimuli-responsive A-SCH/OSA@sEVs microsphere system effectively modulates immune responses, promotes angiogenesis, and enhances osteogenesis, further investigation is needed to deepen mechanistic understanding and advance translational prospects. Given that sEVs carry a wide range of bioactive molecules (e.g., mRNAs, miRNAs, and proteins), the therapeutic effects observed in this study are unlikely to be driven by a single factor. Thus, additional high-throughput analyses (e.g., small RNA sequencing and proteomics) are required to identify the key sEVs cargos involved and to clarify the signaling pathways through which they regulate bone repair. In terms of *in vivo* evaluation, while the microsphere platform showed controlled release, prolonged local retention, enhanced bone regeneration, and good tissue integration, future studies should assess its spatiotemporal performance across multiple stages of bone repair in larger defect models to better capture the dynamic immune-vascular-osteogenic interplay under physiological conditions. Moreover, because the rat calvarial defect is non-load-bearing, mechanical comparisons between regenerated and native bone should be performed in load-bearing bone defect models. Finally, given the bone-targeting property of the A-SCH/OSA microspheres and the superior immunomodulatory, pro-angiogenic, and pro-osteogenic activities of CD301b⁺-sEVs, this strategy may also be effective in more complex pathological bone repair environments beyond the non-pathological model used here. From a translational perspective, the use of FDA-approved alendronate and biocompatible polysaccharides supports safety, while microfluidic fabrication offers scalability and batch-to-batch reproducibility essential for Good Manufacturing Practice (GMP) compliance. The cell-free sEVs platform avoids immunogenicity and storage issues associated with live cells. These features provide a promising foundation for further preclinical development and eventual clinical application. Nevertheless, future translational development will require further optimization of sEVs standardization, dose control, storage stability, and long-term safety of microsphere degradation products.

## Conclusion

Transcriptomic and functional analyses revealed that bioactive cargoes of CD301b⁺- sEVs were distinctly enriched in biological processes related to anti-inflammation, angiogenesis, and osteogenesis, and exhibited superior synergistic regenerative effects compared to conventional M2-sEVs. To overcome the inherent limitations of sEVs therapy, such as short half-life and nonspecific distribution, we engineered a bone-targeted microsphere system (A-SCH/OSA@sEVs). This platform combines three synergistic design principles: (i) ALE mediated HAP targeting for site-specific accumulation and prolonged retention; (ii) pH-responsive Schiff-base hydrolysis for controlled sEVs release triggered by the acidic microenvironment of bone injury; and (iii) coordinated activation of an integrated “immune-vascular-osteogenic” regenerative network. Both *in vitro* and *in vivo* studies confirmed that the system effectively remodels the local bone niche and significantly enhances bone defect repair. Collectively, this work establishes a functionalized sEVs delivery paradigm and proposes a novel regenerative strategy characterized by “targeted localization, smart release, and multi-effect synergy”, underscoring both the scientific innovation of leveraging CD301b⁺-sEVs and its translational potential for bone repair.

## Supplementary Material

Supplementary materials and methods, figures and table.

## Data Availability

Data are accessible upon reasonable request.
