# Supplementary material for: Bone-Targeting Microspheres Enable Sustained Release of CD301b+ Macrophage-Derived Small Extracellular Vesicles to Promote Bone Repair
Source: Theranostics. 2026 Jun 17;16(13):7514–36. doi: 10.7150/thno.132666 (PMC13295118; doi:10.7150/thno.132666)
Supplement: Supplementary file 1 — Supplementary materials and methods, figures and table. [file thnov16p7514s1.pdf]

Supplementary Materials

Bone-Targeting Microsphere Enable Sustained Release of CD301b$^+$ Macrophage-Derived Small Extracellular Vesicles to Promote Bone Repair

Xiang Gao[1,2#], Shanshan Ma[1,2#], Yeying Dong[1,2#], Fengkai Yang[1,2], Rui Huang[1], Xiudan Zheng[1], Zhijun Liu[2], Hong Zheng[2], Fatai Lu[2], Thomas Groth[3], Mingyan Zhao[1,2, 4*]

[1]Stem Cell Research and Cellular Therapy Center, [2] Orthopedic Center, [4]Guangdong Provincial Key Laboratory of Autophagy and Major Chronic Non-communicable Diseases, Affiliated Hospital of Guangdong Medical University, Zhanjiang 524001, China

[3]Department Biomedical Materials, Institute of Pharmacy, Martin Luther University Halle-Wittenberg, 0699 Halle (Saale), Germany

*Corresponding author: Mingyan Zhao, Affiliated Hospital of Guangdong Medical University, Zhanjiang 524001, China. E-mail address: mingyan.zhao@gdmu.edu.cn

[#]These authors contributed equally to this manuscript.

Experimental Sections

BMSCs isolation

Under sterile conditions, the collected bone marrow aspirate was promptly stored in a 50 mL Falcon tube. An equal volume of PBS buffer was gently mixed after dispersing the tissue. Twice the volume of Percoll separation solution (Solarbio life science) was carefully layered beneath the sample, ensuring that the sample remained suspended above the separation medium. Centrifugation was then performed at 400 × g for 20 minutes at 4°C. The cloudy mononuclear cell layer at the interface was carefully aspirated, mixed with 10 volumes of PBS buffer, and then subjected to further centrifugation (250 × g, 10 minutes). After centrifugation, the resulting cell pellet was resuspended in complete medium (α-MEM medium containing 10% FBS and 1% penicillin-streptomycin), transferred to culture flasks, and grown at 37°C in a humidified atmosphere containing 5% CO_2. Upon reaching a confluence of 80~90%, cells were expanded by passage, with medium replaced every three days. Cell status was routinely monitored using an inverted microscope (DMI 3000B, Leica, Germany).

Characterization of BMSCs

BMSCs were harvested via trypsinization, centrifuged, and the resulting cell pellet was resuspended in PBS at a concentration of 1×10^6 cells/mL. Subsequently, the cell suspension was stained with fluorescently conjugated antibodies targeting specific surface markers, namely CD34 (1:400 dilutions, BioLegend, USA), CD45 (1:400 dilutions, BioLegend), CD73 (1:400 dilutions, BD Biosciences, USA), CD90 (1:400 dilutions, BD Biosciences), and CD105 (1:400 dilutions, BD Biosciences). The stained cells were analyzed on a FACSCanto II flow cytometer flow cytometry (BD Biosciences) following the manufacturer's protocols, and the data were processed with FlowJo software (version v10.8.1).

To assess multilineage differentiation potential, BMSCs were separately cultured in three different induction media for 21 days: osteogenic medium for 21 days, adipogenic medium (DMEM supplemented with 10% FBS, 1% penicillin/streptomycin, 50 μM indomethacin, 500 μM 3-isobutyl-1-methylxanthine, 10 μg/mL insulin, and 500 nM dexamethasone, and chondrogenic medium (α-MEM containing 1% penicillin-streptomycin, 1% FBS, 1% ITS, 20 nM dexamethasone, 5 μg/mL L-ascorbic acid, and 10 ng/mL TGF-β1 (Pepro Tech)); all from Sigma unless otherwise stated. Following induction, the osteogenic, chondrogenic and adipogenic differentiation were evaluated using ARS staining, alcian blue staining (OriCell) and oil red O staining (OriCell), respectively.

CD301b$^+$ macrophage isolation by FACS

RAW264.7 cells were induced with 20 ng/mL IL-4 for 72 hours, then collected and prepared as single-cell suspensions. The cells were incubated with F4/80 (1:400 dilutions, BioLegend) and CD301b primary antibodies (1:200 dilutions, BioLegend). After a 30-min incubation at room temperature, the cell-antibody mixtures were subjected to centrifugation at 2,500 rpm for 5 min, and the supernatant was carefully removed. The resulting cell pellet was resuspended in sterile PBS and passed through a 40-μm cell filter. Cell sorting was then conducted on a BD FACSAria II flow cytometer (USA). As a result, CD301b$^-$ and CD301b$^+$ macrophages were obtained and subsequently grown in DMEM complete medium. The sorting efficiency of CD301b$^+$ macrophages was determined by flow cytometry, and data analysis was conducted using FlowJo software.

sEVs isolation and characterization

CD301b$^-$, CD301b$^+$ macrophages, and IL-4-treated RAW264.7 cells (20 ng/mL) were grown in DMEM medium consisting of 10% sEVs-free FBS and 1% penicillin-

streptomycin. To obtain sEVs-free FBS, serum was subjected to ultracentrifugation at 100,000 × g for 12 hours at 4 °C, passed through a 0.22-μm cell strainer, and aliquoted for storage at –80 °C. After 48 h of culture, the culture medium was harvested and sequentially centrifuged at 4 °C to remove dead cells and cellular debris: first at 300 × g for 10 min, then at 2,000 × g for 10 min, and finally at 10,000 × g for 30 min. The resulting supernatant was then subjected to ultracentrifugation at 100,000 × g for 70 min at 4 °C. The obtained pellet was briefly rinsed with chilled PBS, and centrifuged again under the same conditions. The final small extracellular vesicle (sEVs) pellet was then resuspended in PBS, and its concentration was quantified using a BCA kit (Beyotime, China) according to the manufacturer's instructions. The resuspended sEVs, namely $CD301b^-$ macrophages-derived sEVs ($CD301b^-$-sEVs), $CD301b^+$ macrophages-derived sEVs ($CD301b^+$-sEVs), and M2 phenotype macrophages-derived sEVs (M2-sEVs) were kept at -80 °C for subsequent characterization.

The microstructure of the isolated sEVs was visualized using TEM, and their size distribution was detected using NTA. Western blot analysis was applied to verify the expression of canonical surface markers of sEVs (CD63, CD81, CD9 and TSG101) and negative control (Calnexin).

For subsequent cellular uptake experiments, a portion of the sEVs was fluorescently labeled with PKH26. Briefly, 4 μL of PKH26 dye was mixed with 200 μL of Diluent C. This resulting solution was then combined with 100 μL of sEVs suspended in 200 μL of Diluent C. After a10-minute incubation, staining was stopped by adding 500 μL of sEVs-depleted FBS. The mixture was then ultracentrifuged at 120,000 g for 70 min at 4°C in the dark. The labeled sEVs (PKH26-positive) were collected and resuspended in PBS buffer.

Transcriptome sequencing analysis

Briefly, the extraction of RNA was performed using the TRIzol method (Invitrogen). Sequencing libraries were constructed with the NEBNext® Ultra™ RNA Library Prep Kit for Illumina® (NEB, USA). To assess the library quality, Agilent Bioanalyzer 2100 system was utilized, and qualified libraries were sequenced on the Illumina NovaSeq 6000 platform. Raw sequencing reads were mapped to the reference genome using the STAR software. Based on the alignment results, read counts per gene were quantified with HTSeq v0.5.4 p3, and the expression levels of gene were estimated as FPKM values. DEGs were determined using the DESeq R package (version 1.10.1), with screening thresholds set at an adjusted *P*-value (q-value) < 0.05 and $|\log_2 FC| \geq 1$. For the significant DEGs, GO enrichment evaluation was conducted using the GOseq R package according to the Wallenius non-central hypergeometric distribution. Meanwhile, KEGG pathway enrichment was tested with KOBAS software. Finally, a heatmap showing the expression patterns of DEGs was created with the online platform https://www.bioinformatics.com.cn.

Alizarin red S (ARS) staining

BMSCs were plated into 12-well plates at 5×10^4 cells per well. Upon reaching a convergence of 80%, the old medium was discarded and exchanged with OIM, which supplied with either sEVs (M2-sEVs or CD301b$^+$-sEVs, 20 μg/mL or 40 μg/mL), M2 macrophage-conditioned medium (M2-CM) or CD301b$^+$ macrophage-conditioned medium (CD301b$^+$-CM). To prepare the conditioned media, macrophage supernatants were mixed 1:1 with fresh DMEM, followed by supplementation with the aforementioned osteogenic supplements. After co-culturing the cells for 14 days, the degree of mineralization was assessed using ARS staining. Following fixation, the cells were stained with ARS solution for 15 min and then examined under an inverted fluorescence microscope. For the quantification of calcium deposition, the ARS-stained

nodules were dissolved in 10% (w/v) 1-cetylpyridinium chloride monohydrate and measured at 562 nm using a multifunctional microplate reader.

Synthesis and characterization of polysaccharide derivatives

Synthesis and characterization of succinylated chitosan (SCH)

Synthesis of SCH was performed according to our previously published protocol[1], Briefly, Chitosan (CH, 0.5 g) was dissolved in 40 mL of 5% lactic acid aqueous solution (prepared from 85% lactic acid) under magnetic stirring for 4 h. Subsequently, 160 mL of methanol was added, followed by the introduction of 150 mg of succinic anhydride (Sigma) to initiate the succinylation reaction. The reaction proceeded under vigorous stirring for 24 h at room temperature. The resulting mixture was neutralized to pH 7.4 using 10 M NaOH to induce precipitation. The precipitate was collected by filtration, redispersed in 100 mL deionized water and stirred overnight at 800 rpm. The solution was then dialyzed (MWCO: 3500 Da) against deionized water for 72 h, with the water being replaced twice daily. The purified product was lyophilized using a freeze-drier (NingboScientz, China) and stored at 4°C for subsequent use. The chemical structure of SCH was detected using FT-IR.

Synthesis and characterization of oxidized sodium alginate (OSA)

The preparation of OSA was performed according to our previously published protocol[2]. In brief, sodium alginate (SA, 4 g) was added to 20 mL ethanol under magnetic stirring, followed by the addition of sodium periodate solution (molar ratio SA: $NaIO_4$ = 1:0.5, Sigma). The reaction was kept for 6 h under continuous stirring. The mixture was then dialyzed against deionized water (MWCO 3500 Da) for 72 h with water changed twice daily and subsequently lyophilized to obtain OSA. The product was stored at 4 °C until use. Successful oxidation was confirmed by FT-IR analysis through comparison of SA and OSA spectra.

Synthesis and characterization of alendronate-functionalized SCH (A-SCH)

A-SCH was synthesized according to a published literature with minor modifications[3]. In brief, 1.2 g of ALE (Aladdin) was dissolved in 30 mL of 50% glutaraldehyde solution (Macklin) and stirred overnight at 45 °C. After standing for 20 min, the product was washed with a large amount of cold acetone (Macklin), yielding a brown precipitate. The precipitation was thoroughly dried in a ventilated environment to obtain aldehyde-activated ALE. Separately, 250 mg of SCH was added to 150 mL of ultrapure water under stirring until completely dissolved. Then, 50 mg of the aldehyde-activated ALE was introduced and allowed to react at 37°C for 12 h under continuous stirring. The resultant mixture was dialyzed (MWCO: 3500 Da) against deionized water for 72 h and freeze-dried to obtain A-SCH, which was stored at 4°C. Successful grafting of ALE onto SCH was confirmed by ^{31}P nuclear magnetic resonance (^{31}P NMR; JNM-ECS400 from JEOL, Japan), which verified the presence of polyphosphate moiety.

Biocompatibility of microspheres

BMSCs (2×10^3 cells/well), HUVECs (3×10^3 cells/well) and RAW264.7 cells (5×10^3 cells per well) were seeded separately in the lower chambers of 24-well Transwell plates (8 μm pore size, Corning, USA). Microspheres (SCH/OSA, A-SCH/OSA, or A-SCH/OSA@sEVs, 0.5 mg per well) were then placed in the upper chambers, immersed in the corresponding culture media as described in **Section 2.2** of the main manuscript. After 1 and 3 days of culture, the proliferation of cells was determined by the CCK-8 assay, and the absorbance of each well was detected at 450 nm. On day 3, cell viability was evaluated for all three cell types using a live/dead staining kit.

Afterwards, the cytoskeletal organization and cell spreading of BMSCs and HUVECs cultured in the presence of microspheres were examined by CLSM. Briefly, BMSCs and HUVECs were seeded onto confocal dishes (NEST, China) and allowed to adhere

overnight. Microspheres (SCH/OSA, A-SCH/OSA, or A-SCH/OSA@sEVs, 2 mg per dish) were then directly added to the adherent cells in complete medium. After culture for 3 days, cells were briefly rinsed with PBS, fixed in 4% paraformaldehyde for 15 min, permeabilized in 0.1% (v/v) Triton X-100 for 30 min, and blocked in 1% BSA for 60 min. Subsequently, cells were incubated with Alexa Fluor 568 phalloidin (1:40 dilutions, Thermo Fisher Scientific) at ambient temperature for 60 min. Nuclei were counterstained with DAPI (Vector Laboratories, USA) prior to imaging. Cellular morphology and cytoskeletal architecture were comprehensively evaluated using an inverted fluorescence microscope for lower-magnification overview and CLSM with Z-stack acquisition for high-resolution visualization.

RNA isolation and RT-qPCR

Cells were lysed with TRIzol reagent (Invitrogen), and total RNA was isolated following standard phenol-chloroform extraction protocols[4]. The resulting RNA was subsequently converted to cDNA using HiScript III RT SuperMix (Vazyme, China). RT-qPCR analysis was conducted on the ABI Prism 7500 Real-time PCR system. Relative expression levels of the target genes were determined using the $2^{-\Delta\Delta CT}$ method. The corresponding primer sequences are provided in **Table S1**.

Western blot analysis

Total protein from BMSCs or HUVECs were lysed using RIPA lysis buffer (Thermo Fisher Scientific). Aliquots of 30 μg were separated via SDS-PAGE and blotted onto PVDF membranes (Merck Millipore, Ireland). Following a TBST (Solarbio) wash, the membranes were blocked with 5% non-fat milk (Becton, USA) and then incubated overnight at 4°C with primary antibodies against CD63 (1:1000, Zenbio, China), CD81 (1:1000, Zenbio), CD9 (1:1000, Zenbio), TSG101 (1:1000, Zenbio), Calnexin (1:1000, Genuinbiotech), ALP (1:1000, Zenbio), RUNX2 (1:1000, Zenbio), OPN

(1:1000, Proteintech), VEGF (1:1000, Zenbio), HIF-1α (1:1000, Zenbio), CD31 (1:1000, Proteintech), Akt (1:1000, Cell Signaling Technology), p-Akt (1:1000, Cell Signaling Technology), GSK-3β (1:1000, Proteintech), p-GSK-3β (1:1000, Zenbio), β-catenin (1:1000, Abcam) and β-actin (1:2000, Proteintech). After a final series of washes, the membranes were exposed to HRP-conjugated secondary antibodies (1:5000, Proteintech) at room temperature for 60 min, followed by visualization of the immunoreactive protein bands by ECL reagent (Zeta life) and capturing using BG-gdsAUTO720 Gel imaging system (Baygene Biotech Company Limited, China).

Results and discussion

Characterization of BMSCs

As shown in **Figure S3A**, the isolated BMSCs exhibited a typical spindle-shaped morphology. The multidirectional differentiation potential assays revealed the following results: (i) Following osteogenic induction, ARS staining showed distinct calcium nodule formation within the BMSCs **(Figure S3B)**; (ii) Alcian blue staining indicated the generation of blue-stained cartilage extracellular matrix **(Figure S3C)**; and (iii) After adipogenic induction, Oil red O staining demonstrated the presence of numerous lipid droplet vacuoles in the cytoplasm **(Figure S3D)**. Flow cytometry analysis **(Figure S3E)** further confirmed that BMSCs expressed high levels of the MSC markers CD90 (99.9%), CD105 (99.5%), and CD73 (100%), while exhibiting extremely low expressions of CD34 (0.22%) and CD45 (0.24%). Collectively, these results confirm the successful isolation of BMSCs with definitive MSC phenotype, multilineage differentiation capacity, and high purity. They demonstrated osteogenic potential, combined with their established role in bone regeneration, positions these BMSCs as an optimal cell source for subsequent studies.

Osteogenic differentiation of BMSCs under various conditions

To assess the osteogenic potential of macrophage-derive sEVs versus their cellular counterparts, BMSCs were treated with either macrophage-conditioned media (M2-CM, CD301b$^+$-CM), or corresponding sEVs (M2-sEVs and CD301b$^+$-sEVs). ARS staining **(Figure S6A)** revealed calcium nodule formation across all experimental groups. Notably, compared to their respective conditioned media, both M2-sEVs and CD301b$^+$-sEVs exhibited significantly enhanced mineralized nodule formation in a concentration-dependent manner. Strikingly, CD301b$^+$-sEVs at 40 μg/mL demonstrated the most pronounced mineralized nodule formation, establishing this concentration as the optimal choice for subsequent BMSC osteogenic differentiation studies. Quantitative analysis further validated that the osteogenic effects mediated by sEVs were significantly superior to those of soluble factors in conditioned medium **(Figure S6B)**. These findings not only confirm the paracrine-mediated osteogenic regulatory properties of macrophages but also highlight the functional predominance of sEVs as critical mediators of intercellular communication.

***In vitro* biocompatibility of microspheres**

Live/dead staining, CCK-8 assays, and cytoskeletal staining were carried out to further evaluate the cytocompatibility of the microspheres in BMSCs, HUVECs, and RAW264.7 cells. Live/dead staining after 3 days of exposure revealed over 95% viable cells (green fluorescence) in all microsphere groups, with only minimal red fluorescence (dead cells) **(Figure S12A)**. CCK-8 assays further demonstrated a time-dependent proliferation of all cell types. Notably, A-SCH/OSA and A-SCH/OSA@sEVs microspheres significantly enhanced RAW264.7 proliferation at day 3, while BMSCs and HUVECs proliferation showed no intergroup differences **(Figure S12B-D)**. These results consistently demonstrate that the microspheres possess a favorable cytocompatibility, which may be attributed to the inherent biocompatibility

of the base materials—SCH[5] and OSA[6].

Cell adhesion to biomaterials is essential for subsequent cellular events, including proliferation and viability, thereby facilitating bone tissue regeneration and maturation[7]. In this study, phalloidin-based cytoskeletal staining was used to assess the adhesion and spreading of BMSCs and HUVECs after 3 days of culture in the presence of microspheres. To evaluate cell-microsphere interactions, microspheres were directly introduced to pre-adherent cells cultured on confocal dishes. Notably, after overnight incubation, fluorescence imaging showed extensive cell aggregation and adhesion onto the surfaces of all microsphere types, enabling intimate cell-microsphere interfacial contact **(Figure S12E, F)**. Confocal Z-axis imaging further revealed 3D cellular distribution, with cells forming dense adhesions across microsphere surfaces and extending along the spherical contours. The adhered cells in all groups exhibited well-spread cytoskeletons and fully extended morphologies. This favorable adhesion behavior can be largely attributed to the 3D architecture formed by SCH and OSA[8, 9], which provides a biomimetic microenvironment and enhances cell–material interactions through the intrinsic bioactivity of the components.

Supplementary tables and figures

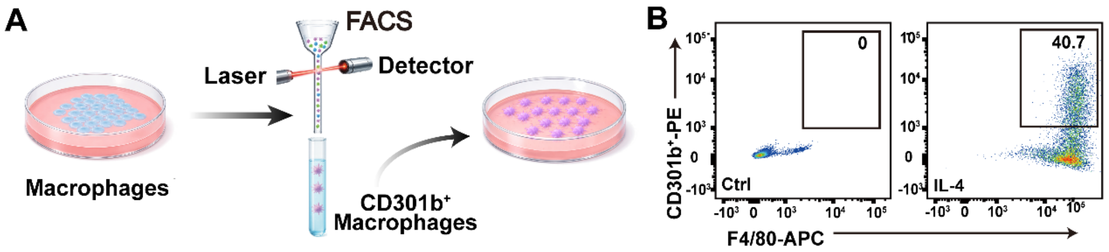

Figure S1. Isolation and characterization of CD301b$^+$ macrophages. (A) Schematic workflow for fluorescence-activated cell sorting (FACS) of CD301b$^+$ macrophages subpopulation. (B) Flow cytometry analysis determining CD301b$^+$ expression in RAW264.7 cells with or without IL-4 induction for 72 h.

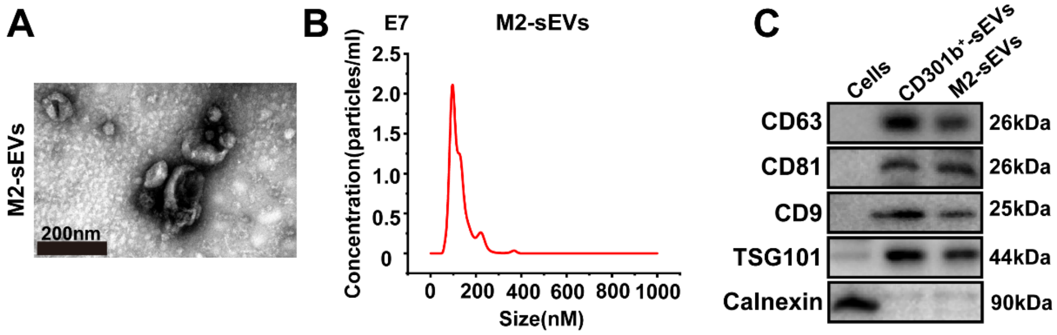

Figure S2. Characterization of M2-sEVs. (A) Representative transmission electron microscopy (TEM) images of M2-sEVs. (B) Size distribution of M2-sEVs detected by nanoparticle tracking analysis (NTA). (C)Western blot analysis determines the presence of sEVs markers (CD63, CD81, CD9 and TSG101) and negative control (Calnexin) in RAW264.7 cells, $CD301b^{+}$-sEVs, and M2-sEVs.

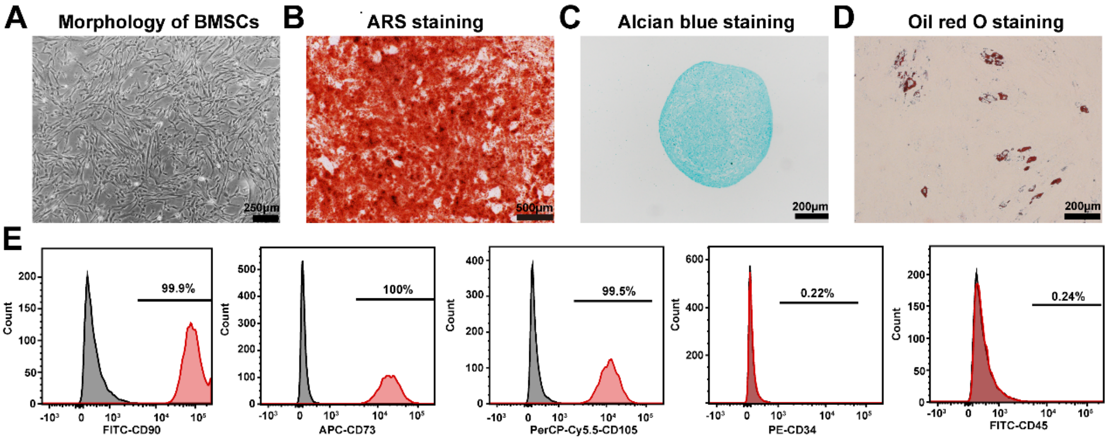

Figure S3. Identification of BMSCs. (A) Morphology of BMSCs after 3 days culture. The multilineage differentiation potential of BMSCs was demonstrated by ARS staining (B), Alician blue staining (C), and Oil red staining (D), respectively. (E) Flow cytometric analysis of BMSCs-specific surface markers revealed that approximately 99% of the cells were positive for CD90, CD73, and CD105, and negative for CD34 and CD45 (less than 0.25%).

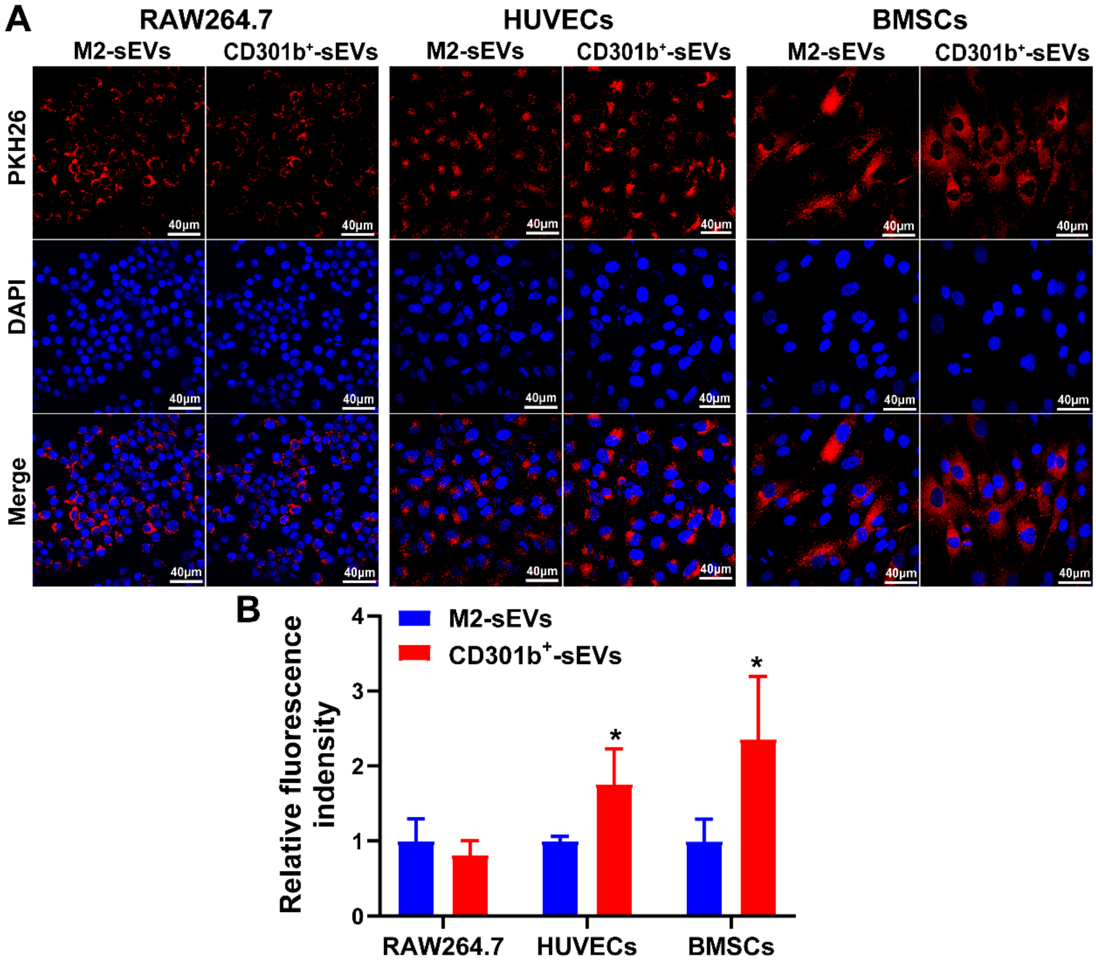

Figure S4. Internalization of PKH26-labeled sEVs by RAW264.7 cells, HUVECs, and BMSCs. (A) Representative fluorescence images of PKH26-labeled sEVs uptake (red); nuclei were counterstained with DAPI (blue). **(B)** Quantitative analysis of sEVs internalization based on fluorescence intensity.

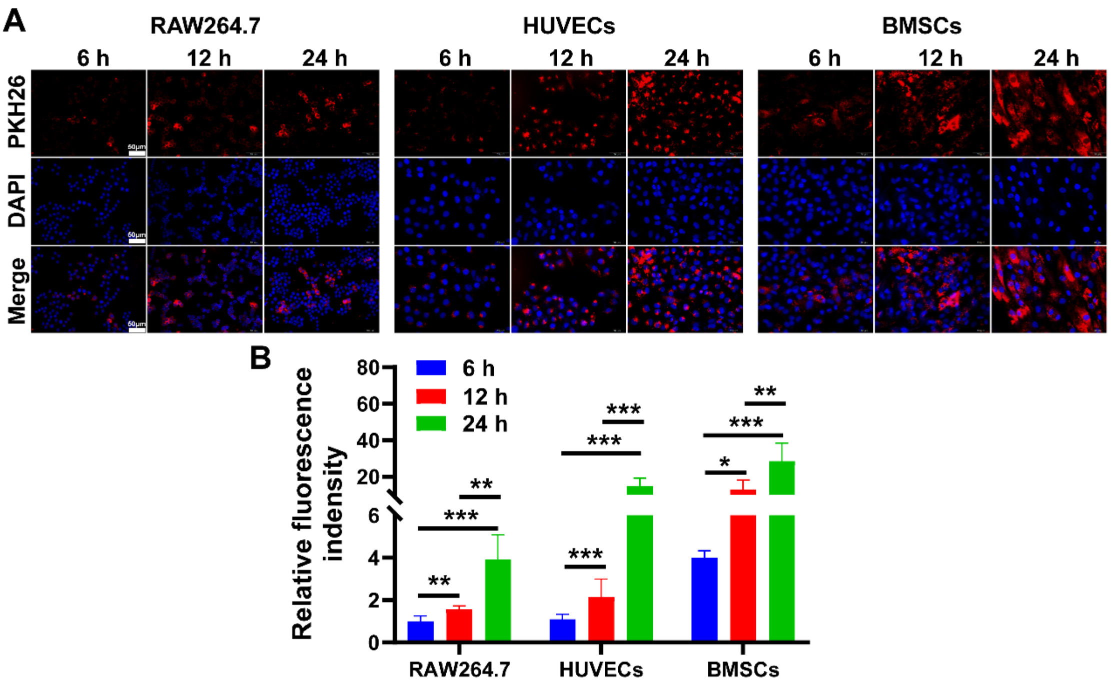

Figure S5. Internalization of CD301b^{+}-sEVs by RAW264.7 cells, HUVECs, and BMSCs. (A) Representative fluorescence images showing the uptake of PKH26-labeled CD301b^{+}-sEVs (red) by RAW264.7 cells, HUVECs, and BMSCs after 6, 12, and 24 h of incubation. Nuclei were counterstained with DAPI (blue). (B) Quantitative analysis of CD301b^{+}-sEVs internalization based on fluorescence intensity.

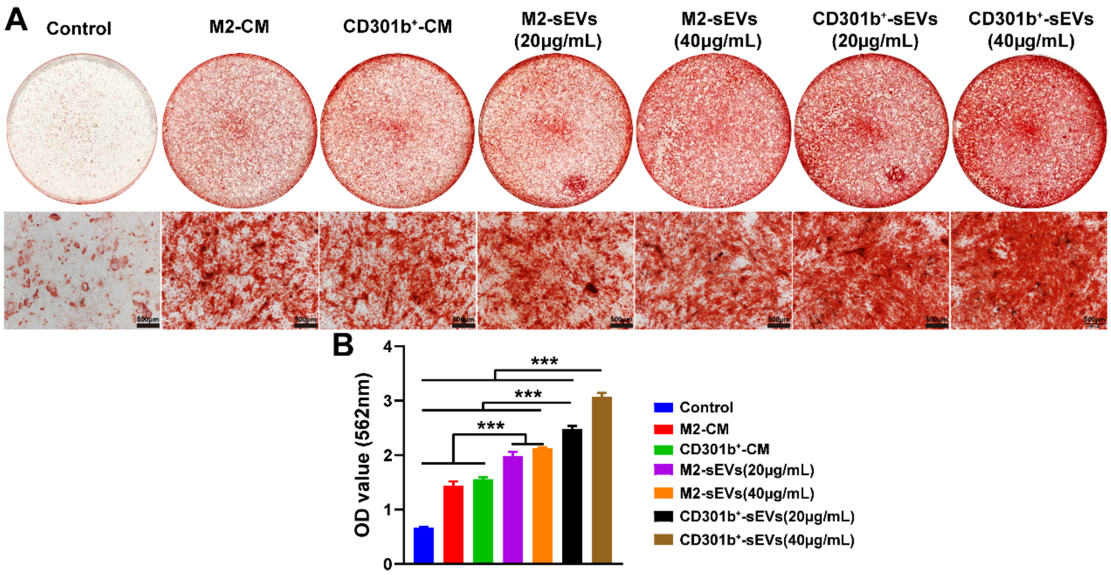

Figure S6. Osteogenic differentiation of BMSCs induced by macrophage-derived conditioned media and sEVs. Alizarin red staining (A) and quantitative analysis (B) determines the mineralization of BMSCs treated with M2-CM, CD301b^{+}-CM, and gradient concentrations of M2-

sEVs or CD301b$^+$-sEVs.

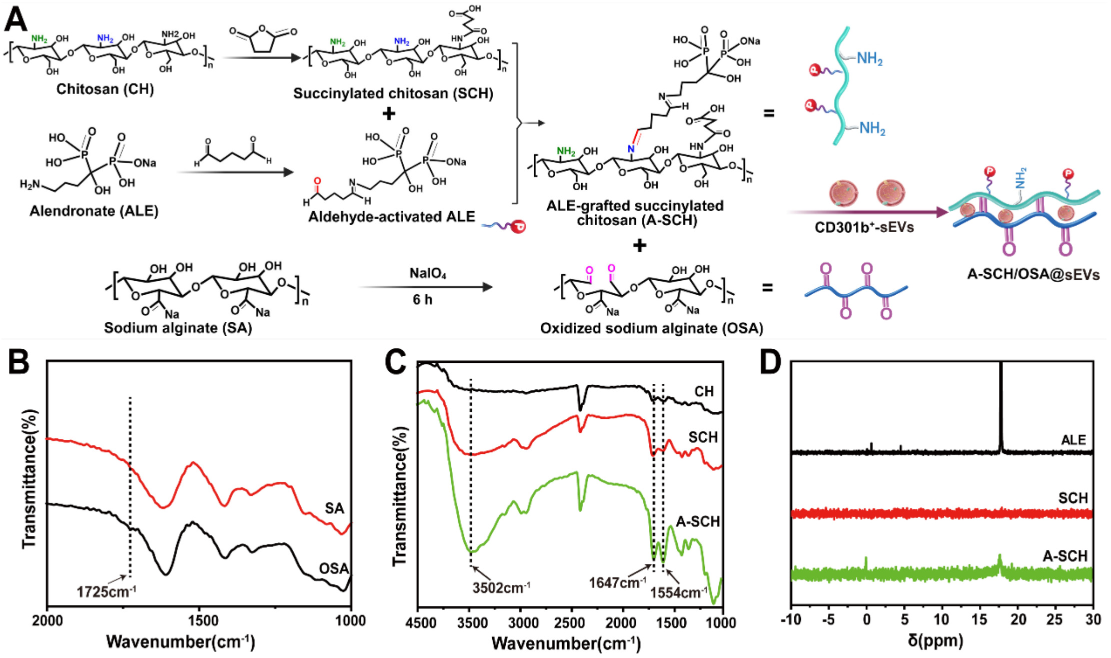

Figure S7. Chemical modification and structural characterization of polysaccharides. (A) Synthesis schemes for the preparation of SCH, A-SCH, and OSA. (B) FT-IR spectra of SA and OSA;(C) FT-IR spectra of CH, SCH and A-SCH; (D) ^{31}P NMR spectra of ALE, SCH and A-SCH.

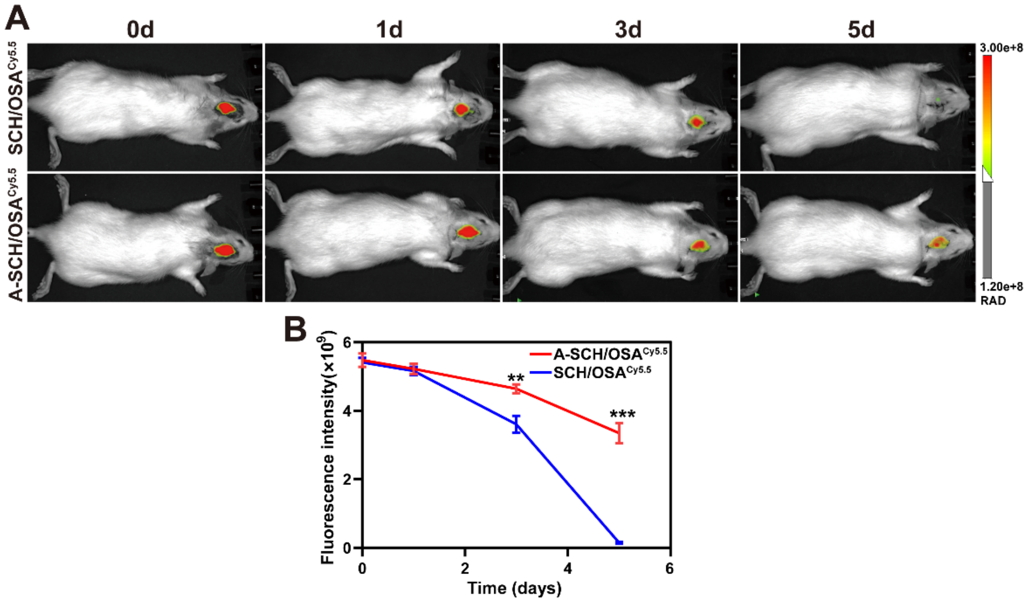

Figure S8. *In vivo* tracking of Cy5.5-labeled microspheres in rat cranial defects (n = 3). (A) Representative near-infrared fluorescence images and (B) corresponding quantitative analysis of fluorescence intensity at the indicated time points.

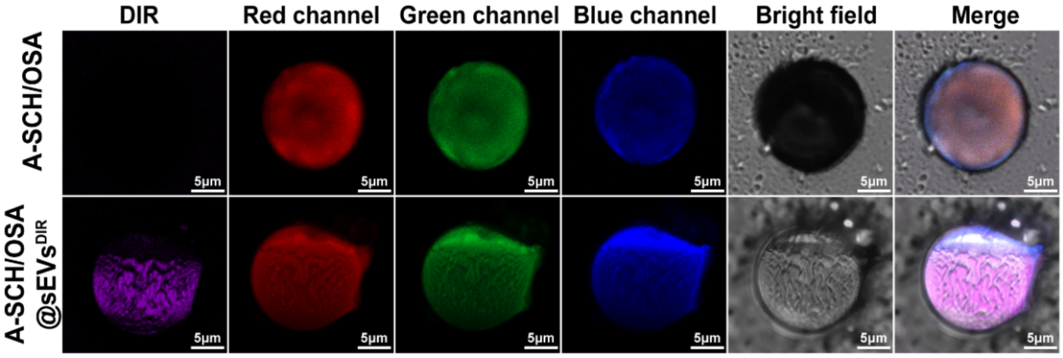

Figure S9. Fluorescence imaging of the cryosections from microspheres loaded with DIR-labeled CD301b^{+}-sEVs.

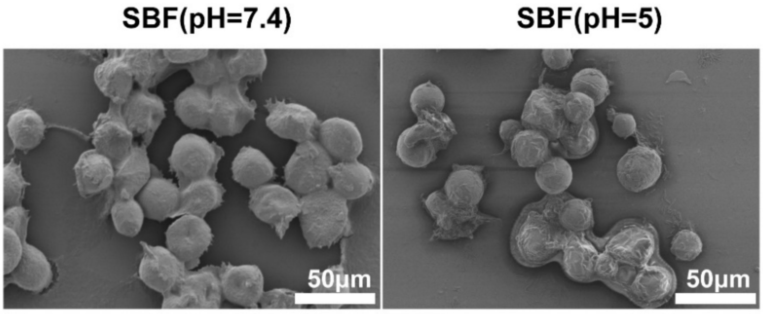

Figure S10. SEM observation of the pH-dependent structural stability of A-SCH/OSA@sEVs microspheres after 15 days of incubation under acidic (pH 5.0) or physiological (pH 7.4) conditions.

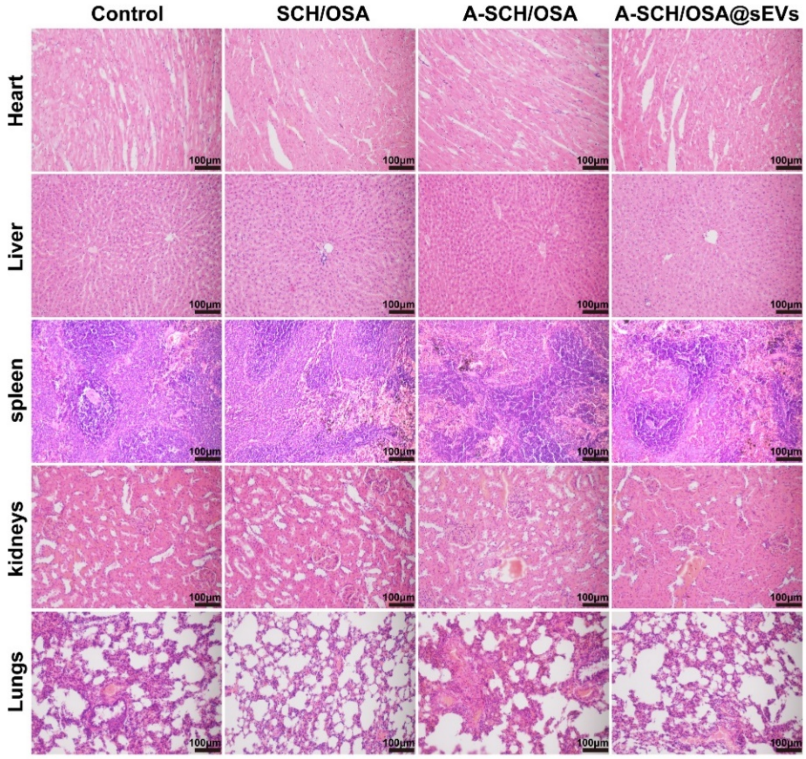

Figure S11. Histopathological evaluation of major organs following long-term microsphere

implantation. Representative H&E-stained images of heart, liver, spleen, kidneys and lungs. [SCH/OSA: plain microsphere fabricated by composition of SCH and OSA; A-SCH/OSA: ALE-modified bone-targeting microsphere composed of A-SCH and OSA; A-SCH/OSA@sEVs: A-SCH/OSA microsphere encapsulated with CD301b^{+}-sEVs; the control (defect-only) group received a saline injection].

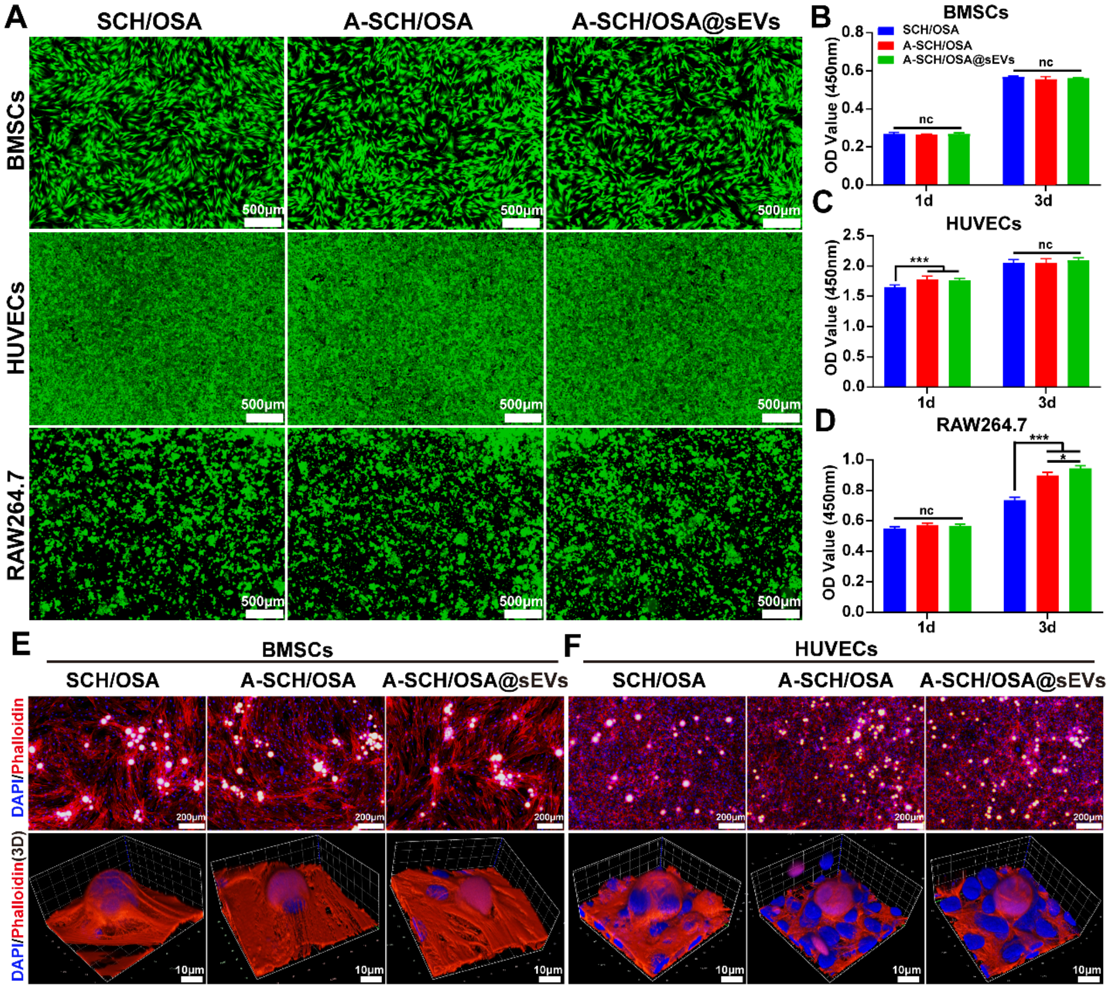

Figure S12. Cytocompatibility analysis of microspheres. (A) Live/dead cell staining of BMSCs, HUVECs, and RAW264.7 cells after 3 days of culture with microspheres (green: live cells, red: dead cells, microspheres were directly added to the culture medium of pre-adherent cells). CCK-8 proliferation assays of BMSCs (B), HUVECs (C), and RAW264.7 cells (D) cultured with different microspheres for 1 and 3 days. CLSM images showing phalloidin-stained cytoskeleton (red: actin) and Z-stack reconstructions of BMSCs (E) and HUVECs (F) cultured in the presence of different microspheres for 3 days. [The microspheres are the same as described in **Figure S11**].

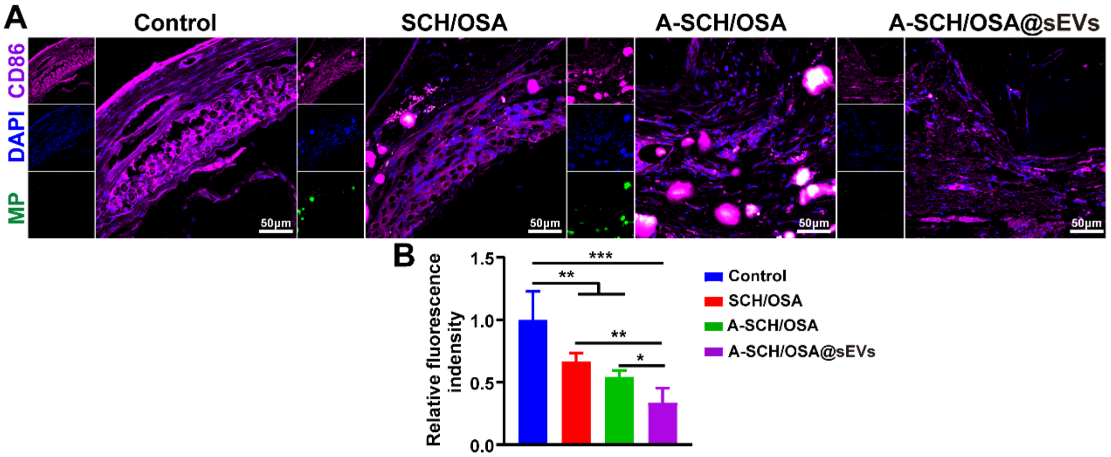

Figure S13. *In vivo* immunomodulatory effects of the different microspheres (MP). (A) Immunohistochemical fluorescence staining detects the expression of macrophage polarization markers CD86 in the defect sites. (B) Quantitative analysis of CD86 expression levels based on fluorescence density. [The microspheres are the same as described in **Figure S11;** the control (defect-only) group received a saline injection].

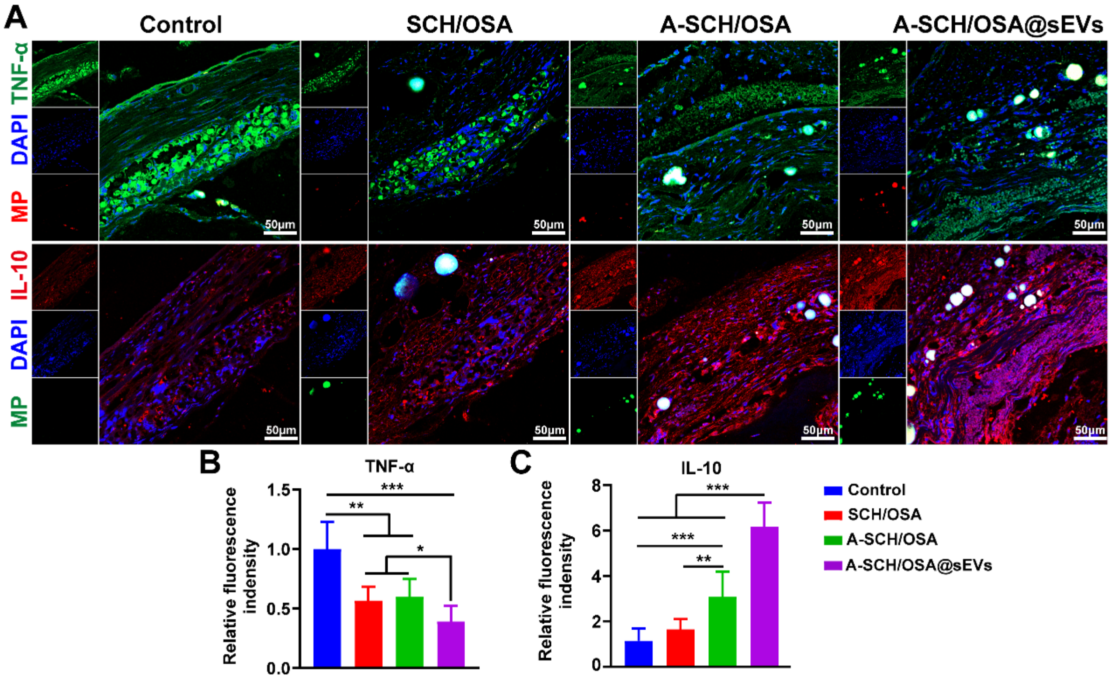

Figure S14. *In vivo* cytokine secretion profiles of the different microspheres. (A) Immunohistochemical fluorescence staining of TNF-α and IL-10 expressions in the defect sites. Quantitative analysis of the fluorescence intensity of TNF-α (B) and IL-10 (C) [The microspheres are the same as described in **Figure S11;** the control (defect-only) group received a saline injection].

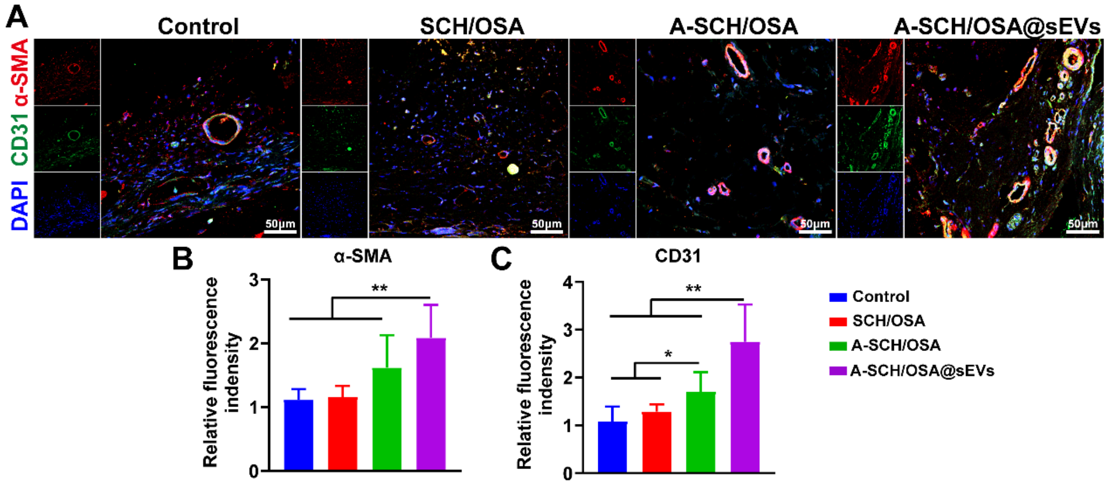

Figure S15. *In vivo* angiogenic potential of the different microspheres. (A) Immunohistochemical fluorescence analysis of angiogenesis by co-staining α-SMA and CD31 expressions in the defect sites. Quantitative analysis of the fluorescence intensity of α-SMA (B) and CD31 (C) [The microspheres are the same as described in **Figure S11;** the control (defect-only) group received a saline injection].

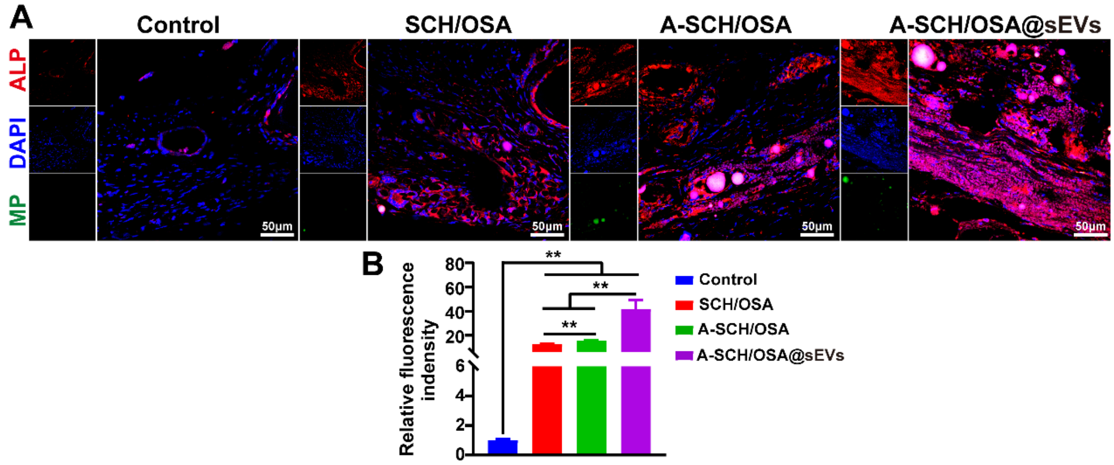

Figure S16. *In vivo* osteogenic potential of the different microspheres (MP). (A) Immunohistochemical fluorescence analysis of bone regeneration by staining ALP expressions in the defect sites. (B) Quantitative analysis of ALP expression levels based on fluorescence density. [The microspheres are the same as described in **Figure S11;** the control (defect-only) group received a saline injection].

Table S1. RT-qPCR primer sequences

Gene	Forward（5'-3'）	Reverse（5'-3'）
RUNX2	CTCTACTATGGCACTTCGTCAGG	TCAGCGTCAACACCATCATTC
ALP	ATGTACTTGGCTCATTGAAGGC	TGCCCCTTATTTCAGGGACTAT
OPN	CTCCATTGACTCGAACGACTC	CAGGTCTGCGAAACTTCTTAGAT
CD206	CTCTGTTCAGCTATTGGACGC	CGGAATTTCTGGGATTCAGCTTC
Arg-1	CCCCATCCACGACAAATGGG	ACCTCTCCATACTGACCGCC
CD86	TGTTTCCGTGGAGACGCAAG	TTGAGCCTTTGTAAATGGGCA
IL-1β	TGGACCTTCCAGGATGAGGACA	GTTCATCTCGGAGCCTGTAGTG
CD31	AGATAACTGAGAGGTTGAGGGAC	ATGGAGCAGGACAGGTTCAGTC
VEGF	AGGGAAAGGGGCAAAAACGA	GAGGCTCCAGGGCATTAGAC
eNOS	GAAGGCGACAATCCTGTATGGC	TGTTCGAGGGACACCACGTCAT
GAPDH	CAGACCACAGTCCATGCCATCAC	GACGCCTGCTTCACCACCTTC

References:

1. Tu C, Gao X, Zheng H, Huang R, Yang F, Dong Y, et al. Innovative injectable, self-healing, exosome cross-linked biomimetic hydrogel for cartilage regeneration. J Control Release. 2025; 381: 113608.
2. Zhao M, Li L, Zhou C, Heyroth F, Fuhrmann B, Maeder K, et al. Improved stability and cell response by intrinsic cross-linking of multilayers from collagen I and oxidized glycosaminoglycans. Biomacromolecules. 2014; 15(11): 4272-80.
3. Zhao Z, Li G, Ruan H, Chen K, Cai Z, Lu G, et al. Capturing Magnesium Ions via Microfluidic Hydrogel Microspheres for Promoting Cancellous Bone Regeneration. ACS Nano. 2021; 15(8): 13041-13054.
4. Dymond J S. Explanatory chapter: quantitative PCR. Methods Enzymol. 2013; 529: 279-89.
5. Chen Q, Qi Y, Jiang Y, Quan W, Luo H, Wu K, et al. Progress in Research of Chitosan Chemical Modification Technologies and Their Applications. Mar Drugs. 2022; 20(8):536.
6. Wu X, Wang Y, Liu X, Ding Q, Zhang S, Wang Y, et al. Carboxymethyl chitosan and sodium alginate oxide pH-sensitive dual-release hydrogel for diabetes wound healing: The combination of astilbin liposomes and diclofenac sodium. Carbohydr Polym. 2025; 349(Pt B): 122960.
7. Farokhi M, Mottaghitalab F, Samani S, Shokrgozar M A, Kundu S C, Reis R L, et al. Silk fibroin/hydroxyapatite composites for bone tissue engineering. Biotechnol Adv. 2018; 36(1): 68-91.
8. He J, Hu X, Cao J, Zhang Y, Xiao J, Peng L, et al. Chitosan-coated hydroxyapatite and drug-loaded polytrimethylene carbonate/polylactic acid scaffold for enhancing bone regeneration. Carbohydr Polym. 2021; 253: 117198.

9. Yu T, Ding Q, Wang N, Zhang S, Cheng Z, Zhao C, et al. Cranial repair-promoting effect of oxidised sodium alginate/amino gelatine injectable hydrogel loaded with deer antler blood peptides. Int J Biol Macromol. 2025; 305(Pt 1): 141116.
